# Inherited Genetic Variation in Parkinson’s Disease: Convergence on Impaired Autophagosome-Lysosome Fusion Through the Altered Expression of mRNA Isoforms

**DOI:** 10.1007/s12035-025-05101-2

**Published:** 2025-06-02

**Authors:** Sreemol Gokuladhas, Catriona Miller, Antony A. Cooper, Justin M. O’Sullivan

**Affiliations:** 1 The Liggins Institute, University of Auckland, Auckland, 1023 New Zealand; 2 Australian Parkinson’s Mission, Garvan Institute of Medical Research, Sydney, NSW Australia; 3 School of Clinical Medicine UNSW Sydney, Sydney, NSW Australia; 4 Maurice Wilkins Centre for Molecular Biodiscovery, Auckland, 1010 New Zealand; 5 MRC Lifecourse Epidemiology Unit, University of Southampton, Southampton, UK; 6 Singapore Institute for Clinical Sciences, Agency for Science, Technology and Research (A*STAR), Singapore, Singapore

**Keywords:** SNCA, α-synuclein, PLEKHM1, Chr17q21.31, Chr16p11.2, Autophagosome-lysosome fusion, Autophagy

## Abstract

Parkinson’s disease (PD) pathogenesis involves complex interactions between genetic factors. We employed two-sample Mendelian randomization (MR) integrating tissue-specific gene regulatory networks to identify causal genes and regulatory elements modulating PD risk. Two-sample MR analysis identified 79 putative causal genes for PD. A subset of the 79 causal genes was enriched within chr17q21.31 and chr16p11.2 cytobands that have been previously linked to neurodevelopmental disorders. Functional enrichment analysis of the 79 genes revealed autophagosome-lysosome fusion as a key process. Ten genes (*ELOVL7*, *HSD3B7*, *PLEKHM1*, *PRSS53*, *SNCA*, *STX1B*, *STX4*, *ZSWIM7*, *LINC02210*, and *RP11-1072 A3.3*) showed causal associations with tissue-specific expression patterns driving risk or protection for PD. Further investigation into their tissue-specific isoform expression profile revealed isoform-specific contributions to disease risk (or protection). These findings highlight the critical role of isoform-specific expression of causal genes in modulating PD risk, particularly relating to autophagosome-lysosome fusion. While our findings provide new insights into PD susceptibility, we acknowledge that the observed isoform-specific changes may, in part, reflect sample selection bias. Therefore, further experimental verification is needed to confirm the importance of incorporating tissue-specific gene isoform profiles in understanding PD causal mechanisms.

**Supplementary Information:**

The online version contains supplementary material available at 10.1007/s12035-025-05101-2.

## Introduction

The hallmark of Parkinson’s disease (PD) is the loss of dopaminergic neurons in the substantia nigra pars compacta, accompanied by the abnormal accumulation of pathological α-synuclein (αSyn) protein aggregates in the central nervous system (CNS). The αSyn protein is produced by the *SNCA* gene, an established risk factor for both Mendelian and sporadic PD [1, 2]. The most abundant *SNCA* transcript encodes the aggregation-prone αSyn-140 AA isoform while additional splice isoforms—e.g. αSyn-126, αSyn-112, αSyn-98, αSyn-66—are also produced by alternative splicing. Previous studies have shown that αSyn isoforms lacking exon5 (αSyn-112 and αSyn-98) are extremely toxic and induce further αSyn aggregation [3, 4]. These αSyn spliced transcript isoforms demonstrate region-specific expression in the brain cortex, substantia nigra, and cerebellum [5].

The aggregation of αSyn within the CNS is a key factor contributing to the motor symptoms of PD, including tremor, bradykinesia, rigidity, and postural instability [6, 7]. However, PD patients often also experience a range of non-motor symptoms such as cognitive impairment, sleep and neuropsychiatric disorders, sensory dysfunction, and gastrointestinal symptoms [8]. Like prions, αSyn can potentially spread to other CNS regions, which might account for its varied deposition patterns in different tissues [9]. This pathological spread of αSyn beyond the CNS into peripheral tissues such as the liver, gastrointestinal (GI) tract, skin, and heart[10, 11] is thought to underlie many of the non-motor symptoms observed in PD. Moreover, PD is progressive in nature and developmental changes that occur in different tissues (e.g. skin and GI tract) could also contribute to the onset of the disease.

Genome-wide association studies (GWAS) have identified about 90 genetic risk loci for PD [12]. However, these susceptibility loci have incomplete penetrance, and the causal genes underlying these association remain elusive. In the last decade, transcriptome-wide association study and two-sample Mendelian randomization (two-sample MR) have been successfully used to identify causal genes by integrating GWAS and tissue-specific gene regulation datasets [13–16]. Critically, the two-sample MR approach does not simply use the top associated SNPs that are identified in GWAS, rather it extensively mines the whole summary dataset to estimate causation.

In this manuscript, we have integrated ‘omic’ datasets (three-dimensional genome structure, expression quantitative loci, population genetic studies [GWAS]) using two-sample MR to identify PD causal genes. Further analysis of the causal genes revealed the central role of autophagosome-lysosome fusion pathway in modulating PD risk. Understanding the inherited genetic variation that impacts the autophagosome-lysosome fusion pathway will help explain apparent contradictions in macroautophagy’s role, as moderate activation degrades damaged organelles, reduces oxidative stress, and promotes neuronal survival, while excessive activation leads to neuronal apoptosis and worsens neurological damage[17]. Moreover, identifying genetic variants impacting autophagic flux will enable targeted therapies that promote autophagosome-lysosome fusion [17, 18] for neuroprotection while distinguishing individuals who would benefit most from these treatments.

## Methods

### Generation of Tissue-Specific Gene Regulatory Networks (GRNs)

The tissue-specific gene regulatory network (GRN) represents a three-dimensional regulatory interaction identified between all pairs of single nucleotide polymorphisms (SNPs) and genes in the tissue of interest. The interaction between a SNP and a gene is considered significant only if a SNP (1) physically interacts with (as informed by Hi-C chromatin contact data) and (2) regulates the expression of its target gene; such SNPs are referred to as spatially constrained expression quantitative trait loci (sceQTLs).

Tissue-specific GRNs for eight tissues (brain cortex, whole blood, artery aorta, artery coronary, liver, lung, skin not sun exposed suprapubic, skin sun exposed lower leg) in the GTEx cohort were generated using matching Hi-C chromatin interaction and expression quantitative trait loci (eQTL) dataset, following the protocol described in [19]. For example, the brain cortex GRN was generated by identifying sceQTL-gene pairs through integration of Hi-C data from dorsolateral prefrontal cortex cells with brain cortex-specific eQTL data from GTEx. Following the discovery of all sceQTL-gene associations, FDR correction was performed to prioritise statistically significant sceQTL-gene associations (FDR < 0.05). Likewise, tissue-specific GRNs were generated for other tissues using respective Hi-C and eQTL data (Supplementary Table 1).

### Identification of PD Causal Genes

Two-sample Mendelian Randomization (two-sample MR) was used to identify genes whose tissue-specific expression changes associated with genetic variants (sceQTLs) confer risk to PD. In this article, PD refers to sporadic PD, unless explicitly stated otherwise. Each genetic variant included in the MR analysis must satisfy three basic assumptions: (i) it is strongly associated with the exposure, (ii) it is not associated with any confounder of the exposure-outcome association, and (iii) it is only associated with the outcome through the exposure. The genetic variant satisfying these assumptions is designated instrumental variable (IV).

Two-sample MR analysis was performed using an R package—‘TwoSampleMR’ (0.5.7) on each tissue independently, using corresponding tissue specific GRN data as an exposure dataset and the PD GWAS[12] as an outcome dataset, as described [19]. Briefly, sceQTLs and their target genes in a GRN with association *p*-value < 1 × 10^−5^ were used as IVs and exposures (or modifiable risk factors) for MR, respectively. All correlated SNPs (*R*^2^ > 0.001; within 10 Mb window) were removed, retaining independent IV(s) for each gene in the exposure dataset. Each IV was searched against the outcome dataset to obtain its effect on PD. When the IV was absent in the outcome GWAS, we substituted it with a proxy SNP (*R*^2^ > 0.9). The IV was excluded from the analysis if a proxy SNP was absent. Importantly, there was no sample overlap between the exposure and outcome datasets, ensuring the validity of the MR assumptions.

After preparing the exposure and outcome datasets, they were harmonised to match the effect alleles in both datasets. Harmonisation was performed using *harmonise_data()* function implemented in the TwoSampleMR package. Briefly, this process ensures that the effect alleles in the exposure and outcome datasets are aligned, palindromic SNPs with intermediate allele frequencies (MAF > 0.42) are removed, strand mismatches are corrected, and allele frequency discrepancies are resolved. Following harmonisation, sensitivity analyses (i.e. heterogeneity and horizontal pleiotropy tests) were performed for genes with more than one IV to exclude IVs with horizontal pleiotropic effects. Finally, MR analysis was performed using the Wald Ratio method if a gene has a single IV; otherwise, inverse variance weighted (IVW) was utilised. The genes with Bonferroni corrected *p*-value less than that of the conservative threshold (*p* < 0.05/number of exposures) are deemed putative causal genes of PD.

### PPMI Transcript and α-Synuclein Seed Amplification Assay (SAA) Data Analysis

Parkinson’s progressive marker initiative (PPMI) is an observational, international, multicenter longitudinal study of people with PD, those at risk for PD (prodromal), and healthy volunteers, aimed at identifying biomarkers of PD progression through clinical, imaging, and genetic data [20]. Detailed information on the PPMI study design, study cohort, participants recruitment can be found in [20]. We collected PPMI transcriptome data from the whole blood samples of PD, prodromal, and healthy control participants according to the PPMI study protocol (https://www.ppmi-info.org/access-data-specimens/download-data). Transcript quantification (TPM) data generated by Salmon was obtained from the PPMI database. Sample collection for RNA-seq library preparation and sequencing, data processing, and quality control is detailed in [21].

The prodromal participants, as classified by PPMI, are 60 years or older and include carriers of monogenic mutations (e.g. *LRRK2*, *GBA*, *SNCA*), or are prodromal with hyposmia (HPSM) or REM sleep behaviour disorder (RBD). Transcript analysis was conducted on data from 416 participants diagnosed with PD [genetic subgroup; *n* = sporadic (221), *GBA* (45), *LRRK2* (131), *SNCA* (19)], 306 individuals in the prodromal stage [genetic subgroup; *n* = HPSM (25), RBD (38), *GBA* (81), *LRRK2* (156), *SNCA* (6)], and 191 healthy controls. The participants had a mean age of 61 ± 10 years that included 44.3% females (*n* = 404) and 55.7% males (*n* = 509). Only participants with available transcript-level TPM data were included in this analysis. We mapped the transcript identifiers to the gene identifiers using gencode genes (v29) and filtered for two *SNCA* transcripts ENST00000420646.6 and ENST00000506691.1, which encode the α-Syn-112 AA and α-Syn-66 AA proteins, respectively. We focused on these two of *SNCA* because they encode less abundant α-Syn proteins, which may play a crucial role in α-Syn aggregation dynamics. Similarly, we obtained the transcript information for *PLEKHM1*. The Wilcoxon test was used to compare differences in the mean transcript expression levels across the PD and prodromal patients.

α-Synuclein seed amplification assay data were obtained from the PPMI for participants at baseline. The SAA categorised participants as positive or negative for αSyn aggregation in the cerebrospinal fluid. Using the whole genome VCF files of the participants for whom SAA data was available, we identified the carriers and non-carriers of SNCA expression modifying risk alleles of SNPs (rs2583990_GG − rs356224_GG) in both SAA + and SAA − groups. A proportion test was performed to compare differences between SAA + and SAA − participants who carried the risk alleles and those who did not. Fisher’s exact test was used to compare differences across groups (carriers and non-carriers).

### Functional Annotation of the Putative Causal Genes of PD

Gene ontology (GO) enrichment analysis was performed to identify ontology terms enriched for putative causal genes of PD. The R package Gprofiler2 [22] was used to perform the enrichment analysis, using all annotated genes in the GO database as a background set. To adjust for multiple testing, *p*-values were corrected using the false discovery rate method. Only GO terms with an adjusted *p*-value (< 0.05) were reported. Furthermore, the ToppGene Suite [23] (https://toppgene.cchmc.org/prioritization.jsp) was utilised to analyse cytoband enrichment among the causal genes. Significant enrichment was determined using a Bonferroni-corrected *q*-value threshold of < 0.05.

## Results

### Two-Sample Mendelian Randomization Identified Putative PD Causal Genes in Eight Tissues

Neurodegeneration is central to PD. However, growing evidence suggests that understanding PD risk requires a holistic approach that considers contributions from other tissues via immune, vascular, and metabolic pathways [24–26]. Therefore, we selected eight tissues with known or potential relevance to PD for the two-sample Mendelian Randomization*** (***two-sample MR) analysis. The adult brain cortex was included due to its neurobiological relevance, while the whole blood and lung were selected for their roles in immune function [27] and environmental exposure [28], both implicated in PD risk. The aorta and coronary arteries were included due to emerging links between vascular dysfunction and PD [24], and the liver was considered for its metabolic roles [10], which have been associated with neurodegeneration.

**Fig. 1:**
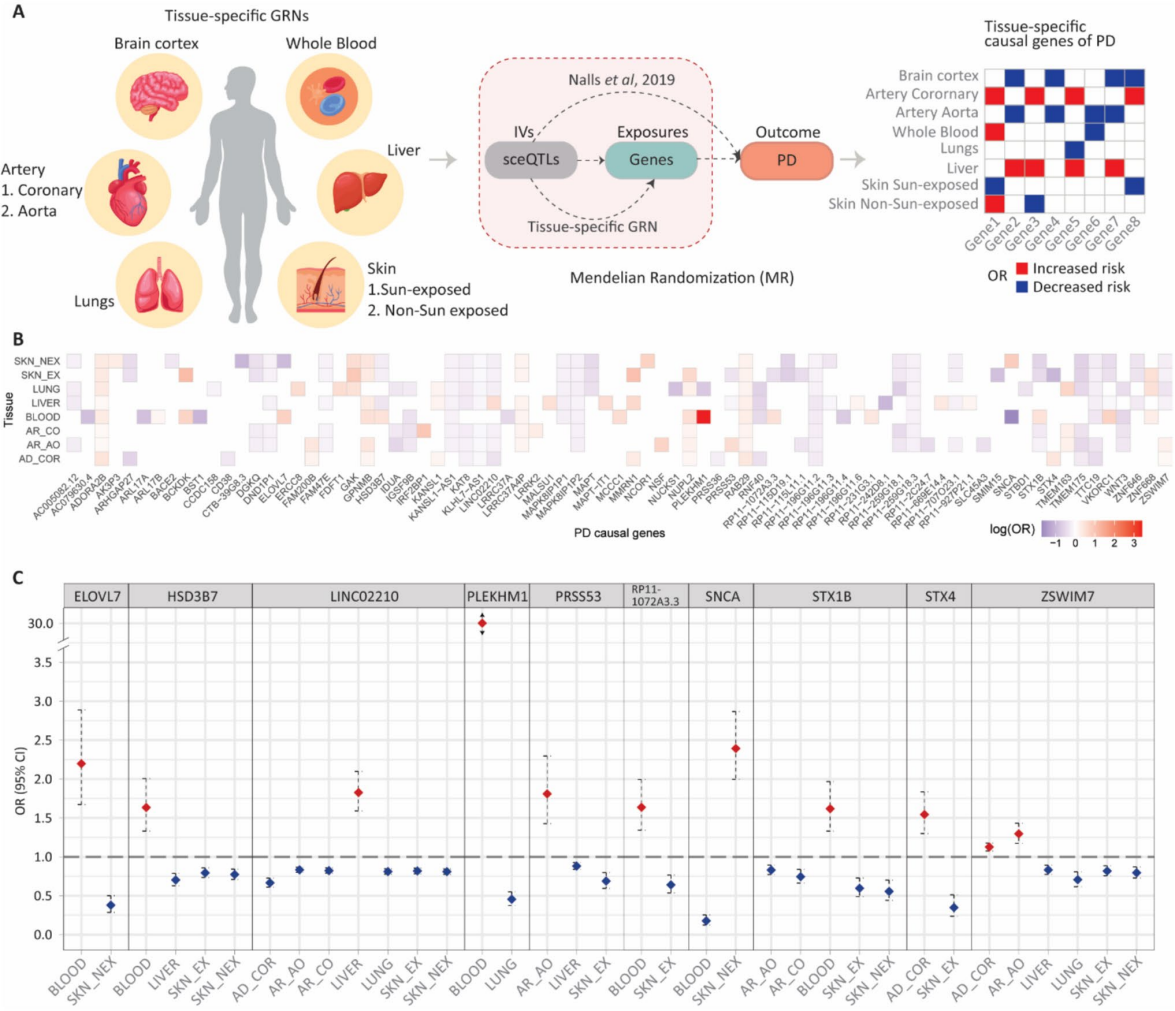
Tissue-specific GRNs inform identification of causal genes of PD. **A** Cartoon outlining method employed in this study. sceQTLs and their target genes identified within eight tissue-specific gene regulatory networks with association *p*-values (< 1 × 10^−5^) were used as IVs and exposures, and the GWAS summary statistics [12] were used as outcome dataset for two-sample MR analysis. Two-sample MR analysis identified genes whose expression changes conferred risk (odds ratio; log (OR) > 0) and protective (log (OR) < 0) effects on PD. **B** Two-sample MR identified 79 putative causal genes for PD across eight tissues. **C** Ten PD causal genes were identified that have both risk and protective effects in different tissues. AD_COR, adult brain cortex; AR_AO, artery aorta; AR_CO, artery coronary; SKN_EX, skin sun-exposed lower leg; and SKN_NEX, skin not-sun-exposed suprapubic

Additionally, including sun-exposed and non-sun-exposed skin tissues was motivated by evidence of peripheral α-synuclein aggregation and molecular changes in PD patients’ skin [29]. Together, these tissues provide a broader systemic perspective on PD pathogenesis. Therefore, we generated tissue-specific gene regulatory networks (GRNs) of these eight tissues (see methods; Supplementary Tables 2–9) and performed two-sample MR, using a meta-GWAS [12] to identify putative causal genes for PD (Fig. 1A). In the two-sample MR experiment, sceQTLs and genes within each tissue specific GRN were used as instrumental variables (IVs) and exposures, respectively, to estimate the effect of gene expression on PD outcome. Odds ratios (OR) from the two-sample MR analysis were used to determine if variation in a gene’s expression was associated with risk (OR > 1) or protection for PD (OR < 1; Fig. 1A). We identified 79 putatively causal genes for PD across the eight tissues (Fig. 1B). OR for causal genes ranged from 30.23 (*PLEKHM1*; 95% CI = 14.79–61.795) to 0.18 (*SNCA*; 95% CI = 0.123–0.253) (Supplementary Table 10). Consistent with earlier reports, *SNCA*, *LRRK2*, and *MAPT* were identified as being causally related to PD [30–32]. Furthermore, a subset of the 79 causal genes was enriched within chr17q21.31 and chr16p11.2 cytobands that have been previously linked to neurodevelopmental disorders (Supplementary Table 11).

Most of the causal genes showed consistent directions of effect (log (OR) > 0 or < 0) across multiple tissues (Supplementary Table 12), highlighting the role of shared genetic architecture and common gene regulatory mechanisms across tissues. However, ten genes *ELOVL7*, *HSD3B7*, *PLEKHM1*, *PRSS53*, *SNCA*, *STX1B*, *STX4*, *ZSWIM7*, *LINC02210*, and *RP11-1072 A3.3* were associated with increased or decreased PD risk (i.e. they had opposite effects) in a tissue specific manner (Fig. 1C; Supplementary Table 12). This finding suggests that these genes may be differentially expressed across tissues, with the associated sceQTLs contributing to tissue-specific regulatory mechanisms.

### The Causal Genes Identified by MR Converge on Autophagosome-Lysosome Fusion

Gene functional enrichment analysis of the causal genes identified by two-sample MR, including those that showed opposing ORs (e.g. *PLEKHM1*), identified enrichment in vesicle-mediated transport in synapse (*p* = 2.27e-02), positive regulation of receptor recycling (*p* = 3.44e-03), vesicle fusion (*p* = 1.65e-02), synaptic vesicle exocytosis (*p* = 2.79e-02), synaptic vesicle priming (*p* = 3.00e-02), process utilizing autophagic mechanism (*p* = 4.95e-02), and autophagy (*p* = 4.95e-02) (Supplementary Table 13). *PLEKHM1*, *SNCA*, *STX1B*, *PRSS53*, *HSD3B7*, *LRRK2*, *GPNMB*, *DGKQ*, *TMEM163*, *TMEM175*, and *IDUA* are all recognised as being involved in autophagosome-lysosome fusion (Table 1). Notably, *PLEKHMI* is associated with the largest increase in OR for PD (Supplementary Table 10) and has an essential role in autophagosome-lysosome fusion, interacting with the HOPS tethering complex and autophagosome membranes. Similarly, *SNCA*, *STX1B*, *PRS533*, *HSD3B7*, *LRRK2*, and *GPNMB* all increase the OR for PD and a) affect autophagosome-lysosome fusion through effects on another factor/stage of the process (*SNCA*, *STX1B*, *HSD3B7*, *PRSS53*, and *LRRK2*)*,* or directly affect the lysosome (*GPNMB*).

**Table 1:** MR identified PD causal genes and their contributions to autophagosome–lysosome fusion

MR			Autophagosome-lysosome fusion	
**Gene**	**No of tissues**	**Have diverging effect on PD**	**Notes**	**Ref**
*PLEKHM1*	2	Yes	Cytosolic adapter protein that coordinates endocytic trafficking and facilitates autophagosome-lysosome fusion	[33]
*SNCA*	2	Yes	α-syn disrupts autophagosome-lysosome fusion by impairing SNARE proteins (e.g. SNAP29) and ykt6, leading to defective clearance of toxic proteins and organelles in PD	[34]
*STX1B*	6	Yes	The SNARE complex, consisting of syntaxin-1B, SNAP25, and synaptobrevin, mediates the calcium-dependent fusion of synaptic vesicles with the presynaptic membrane for neurotransmitter release. Neurotransmitter release and autophagosome-lysosome fusion are interconnected processes at the presynaptic terminal	[35]
*PRSS53*	3	Yes	Involved in the process of autophagosome-lysosome fusion, which is a crucial step in autophagy. Binding partner of INPP5E which is key regulator of fusion. INPP5E regulates lipid composition	[36, 37]
*HSD3B7*	5	Yes	27-hydroxycholesterol (27-OHC) impairs the fusion of autophagosomes with lysosomes, thereby inhibiting autophagic flux and leading to the accumulation of autophagosomes	[37]
*VKORC1*	4	Yes	Vitamin K hydroquinone induces autophagic cell death in cancer cells by triggering metabolic stress and the AMPK pathway, leading to the formation of autophagosomes	[38]
*DGKQ*	4	No	DKGQ negatively regulates the terminal step of autophagy by interacting with VPS39 and preventing the recruitment of the HOPS tethering complex to autophagosomes, thereby blocking their fusion with lysosomes and subsequent cargo degradation	[36]
*TMEM163*	3	No	TMEM163 forms a potassium-permeable leak-like channel that regulates lumenal pH stability and is required for autophagosome-lysosome fusion	[39]
*TMEM175*	5	No	TMEM175 is a lysosomal ion channel that regulates lysosomal membrane potential, pH stability, and ion homeostasis within lysosomes	[40]
*WNT3*	4	No	Wnt signaling acts as a negative regulator of autophagosome-lysosome fusion and autophagic degradation by modulating mTOR activity, TFEB localization, and targeting autophagy proteins for degradation	[41]
*MAPT*	3	No	MAPT accumulation impairs autophagosome-lysosome fusion by downregulating IST1 and disrupting the ESCRT-III complex formation	[42]
*IDUA*	4	No	Depletion of the drosophila homolog of IDUA (referred to as D-idua) leads to defective autophagosome-lysosome fusion	[43]
*GPNMB*	5	No	Overexpression of *GPNMB* in cells leads to impaired fusion of autophagosomes with lysosomes. GPNMB involved in the recruitment of the autophagy protein LC3 to phagosomes	[44]
*LRRK2*	1	No	Hyperactivation of LRRK2 causes defects in transport and maturation of neuronal autophagosomes, establishing autophagy as a key pathway in PD pathogenesis	[45]

### SNCA sceQTLs Were Associated with Both Increased Risk and Protection

**Fig. 2:**
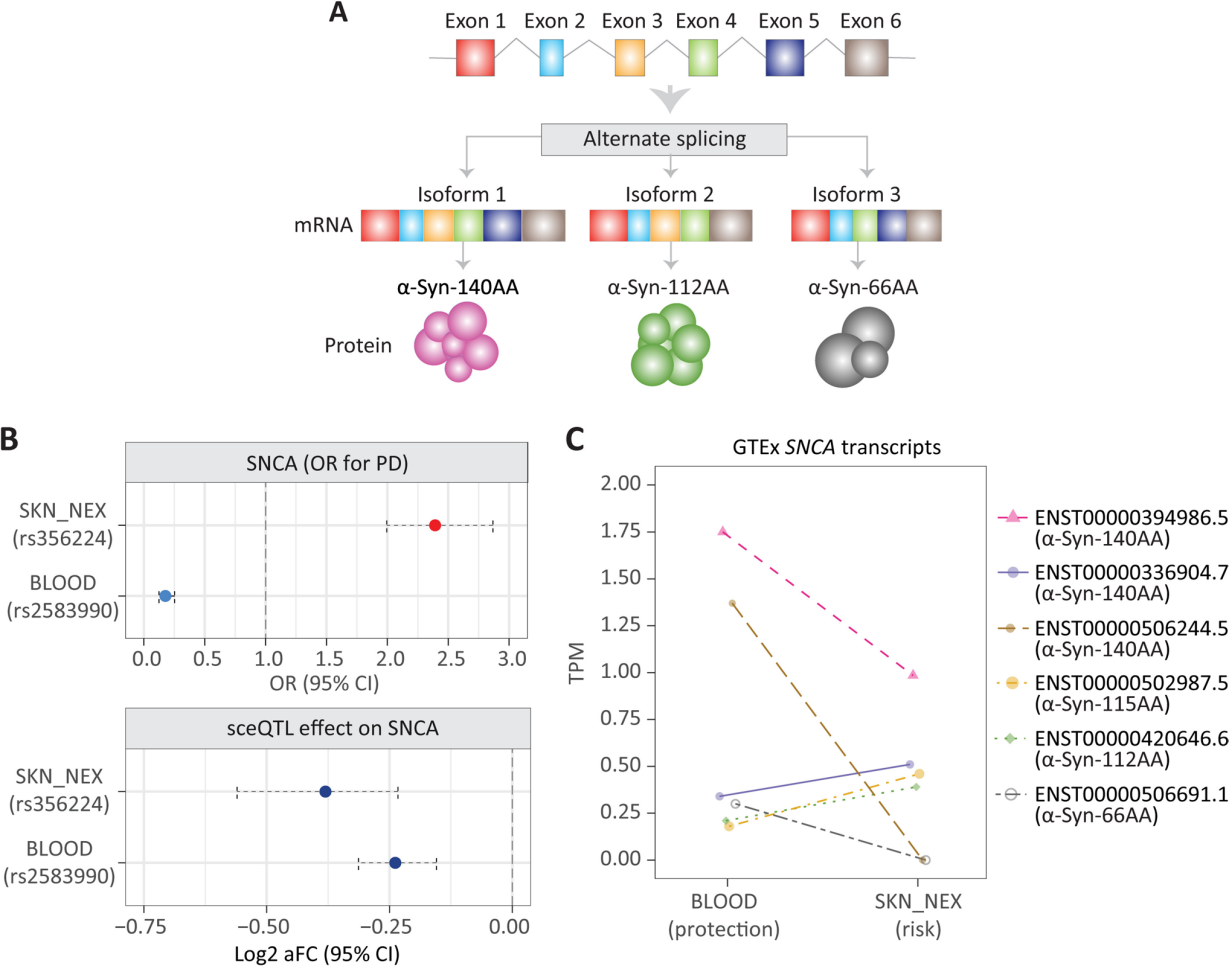
MR estimated causal effect of *SNCA* may be attributed to *SNCA* transcript expression patterns in the blood and skin not-sun-exposed tissues. **A** Schematic representation of *SNCA* and a subset of its isoforms. **B** OR and allelic fold change of *SNCA* expression associated with rs2583990 and rs356224 in blood and SKN_NEX tissues. **C** *SNCA* transcript expression (TPM) levels in blood and SKN_NEX tissues in GTEx samples (Data accessed on 06–12–2023)

*SNCA* is widely recognised as being causally associated with PD. Alternative splicing of the *SNCA* gene results in different mRNA isoforms encoding α-synuclein proteins of varying lengths and exon composition (Fig. 2A). Two-sample MR identified *SNCA* as being associated with increased risk in skin (SKN_NEX) and protection in blood, respectively. SNPs rs356224 (sceQTL in SKN_NEX) and rs2583990 (sceQTL in BLOOD) flank either side of *SNCA* (GRCh38.p14, chr4:89,724,099-89,838,304) and are separated by 127,876 bp. Notably, rs356224 (chr4:89722472 [GRCh38.p14]) and rs2583990 (chr4:89850348 [GRCh38.p14]) are both associated with a decrease in the *total* expression of the *SNCA* gene (Fig. 2B). However, rs356224 is associated with PD risk (OR=1.74 ± 1.41, *p*=1.22 × 10^−7^) while rs2583990 is associated with protection (OR=0.177 ± 0.12, *p*=2.6 × 10^−21^) (Fig. 2B).

### Alterations in SNCA Odds Ratio Are Associated with Altered α-Syn Isoform Specific Transcription and Aggregation

We hypothesized that the SNPs rs356224 and rs2583990 act to modify PD risk by altering expression of specific *SNCA* isoforms. *SNCA* can be transcribed into at least 16 mRNA isoforms encoding proteins of varying length (66–159 AA) (Fig. 2A). Notably, several distinct mRNA isoforms encode α-Syn-140 AA but differ in their 3′ and 5′ untranslated regions. We hypothesized that the observed switch between increased risk (OR > 1) and decreased risk (OR <1) for genes such as *SNCA* is linked to the differential abundance of transcript isoforms in specific tissues (Fig. 2B). Consistent with this hypothesis, the switch from protection to risk-associated *SNCA* expression involves a total loss of the transcript isoforms encoding α-Syn-66 AA and α-Syn-140 AA (ENST00000506224.5) (Fig. 2C). Concurrently, there is a notable increase in other transcript isoforms encoding α-Syn-140 AA (ENST00000336904.7), α-Syn-115 AA, and α-Syn-112 AA, showing increases of 1.5-fold, 2.55-fold, and 1.86-fold, respectively (Fig. 2C). While isoform expression differences and *SNCA* transcript loss are observed, the link to sceQTLs remains a hypothesis, and further experiments are needed to determine underlying mechanisms driving these transcriptional changes.

**Fig. 3:**
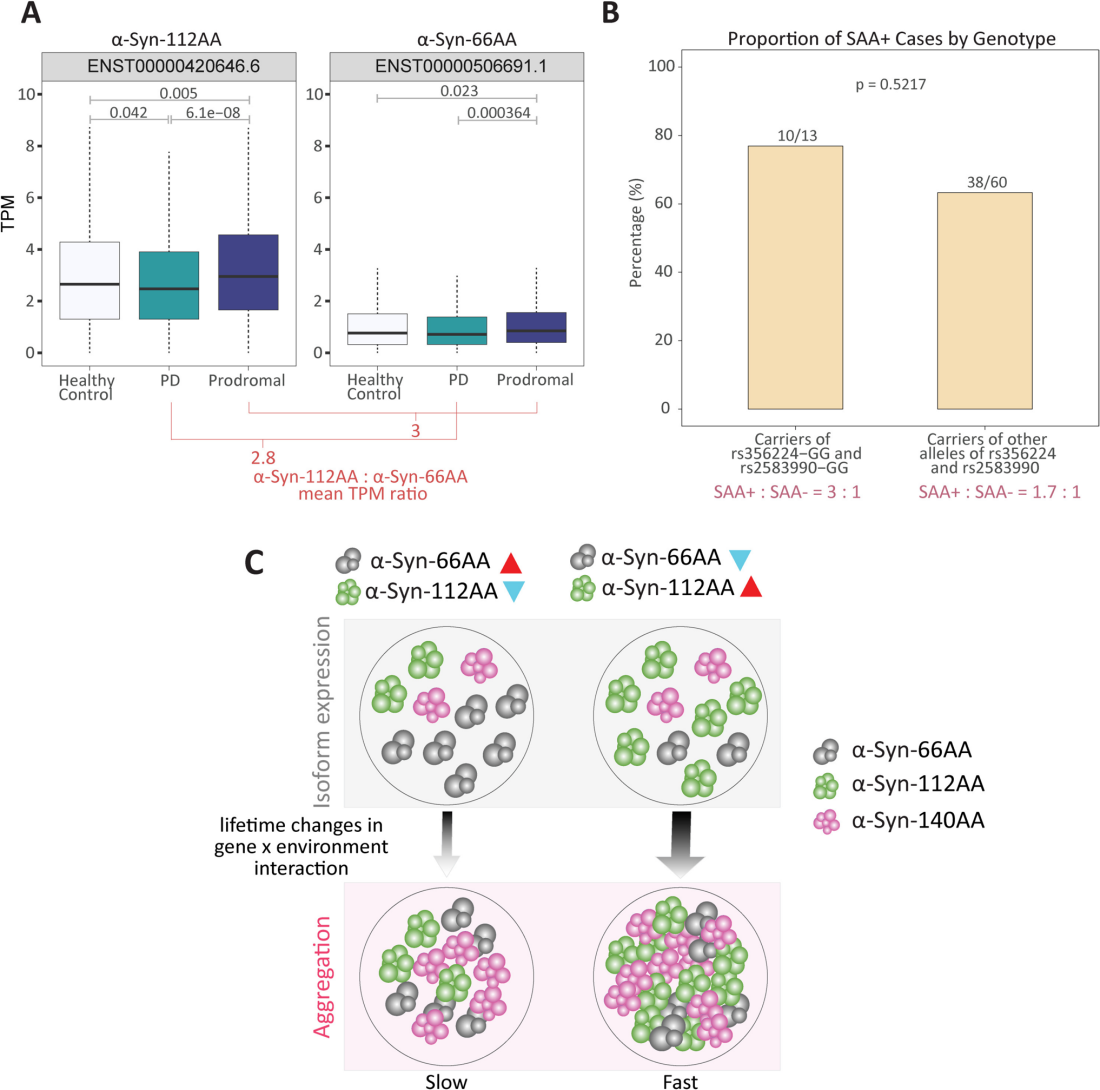
Tissue-specific sceQTLs in *SNCA* may increase α-synuclein aggregation risk by altering isoform transcription. **A** Comparison of transcript expression levels for the α-Syn-112 AA (ENST00000420646.6) and α-Syn-66 AA (ENST00000506691.1) isoforms identified a minor change in the ratio between PD and prodromal patients within the PPMI cohort. **B** Carriers of both the rs356224-GG and rs2583990-GG PD risk variants have a higher frequency of SAA + (α-Syn aggregation) compared to those lacking the risk variants at SNPs rs356224 and rs2583990. **C** Inherited genetic variation in the relative levels of α-Syn-112 AA:α-Syn-66 AA may alter the aggregation rate of αSyn over a person’s lifetime

We observed significant elevated expression of the transcripts encoding α-Syn-112 AA and α-Syn-66 AA in prodromal cases when compared to healthy control and PD patients within PPMI cohort (Fig. 3A). Notably, the prodromal group includes individuals with monogenic mutations (*LRRK2*, *GBA*, *SNCA*).. Similarly, the PD cohort (*n* = 416) also includes both idiopathic and monogenic PD cases, with approximately 195 individuals carrying monogenic mutations. When the analysis was restricted to sporadic PD cases, the expression pattern for these isoforms persisted; however, the difference in ENST00000506691.1 expression was no longer statistically significant, while ENST00000420646.6 remained significantly elevated in the prodromal group compared to sporadic PD. Genetic PD cases showed significant differences in these two isoform expressions compared to healthy controls (Supplementary Fig 1). It remains possible that the lack of significant difference between the healthy controls and sporadic PD patients for α-Syn-112 AA and α-Syn-66 AA may reflect the biological heterogeneity of sporadic PD, where transcript changes are more subtle and not driven by known mutations. Interestingly, the consistent ratios of these isoforms in sporadic and genetic PD patients (Fig 3A; Supplementary Fig 1), compared to prodromal suggest that isoform expression may also be influenced by inherited genetic variants regulating *SNCA* gene. These findings support the hypothesis that isoform expression differences reflect underlying genetic regulatory mechanism rather than being part of a feedback loop accelerating PD progression. The observed association between *SNCA* genetic variants and isoform expression raises the question of how rs356224 and rs2583990 influence transcript levels. While the precise molecular mechanism remains unclear, these SNPs act as sceQTLs and may impact promoter activity, splice site selection, or chromatin conformation, thereby modulating transcript abundance in a tissue-specific manner. Alternatively, given that the *SNCA* locus is subject to structural variation, including inversions, it is possible that these variants may result from the act of inversion and thus are indicators of the active promoter, choice of which determines the isoform-specific expression levels. Further studies are required to experimentally validate these hypotheses and determine the mechanistic link between genetic variation and SNCA transcript isoform regulation.

Empirical evidence has shown that small amounts of α-Syn-112 AA isoform accelerate SNCA aggregation rates [46], underscoring the importance of isoform levels in different tissues, consistent with previous studies [47]. Our analysis showed that a greater percentage of PD patients homozygous for both SNCA risk alleles (rs356224_GG and rs2583990_GG) had a higher α-Syn seed amplification assay (SAA) positive:negative ratio compared to those without these risk alleles (Fig. 3B). While the SAA data is limited by sample size, these findings suggest that *SNCA* isoform expression patterns significantly impact PD risk. Based on these findings, we propose a model where the interplay between genetic predisposition, gene isoform-specific expression, and environmental factors contribute to PD pathogenesis. Specifically, genetic variants regulate the relative expression of transcripts encoding α-Syn-112 AA and α-Syn-66 AA. The altered balance of these isoforms, when interacting with environmental signals, may accelerate α-Syn aggregation over a person’s lifetime, thereby contributing to PD progression (Fig. 3C).

### PLEKHM1 Is the Causal Gene Associated with the Greatest Risk for PD

**Fig. 4:**
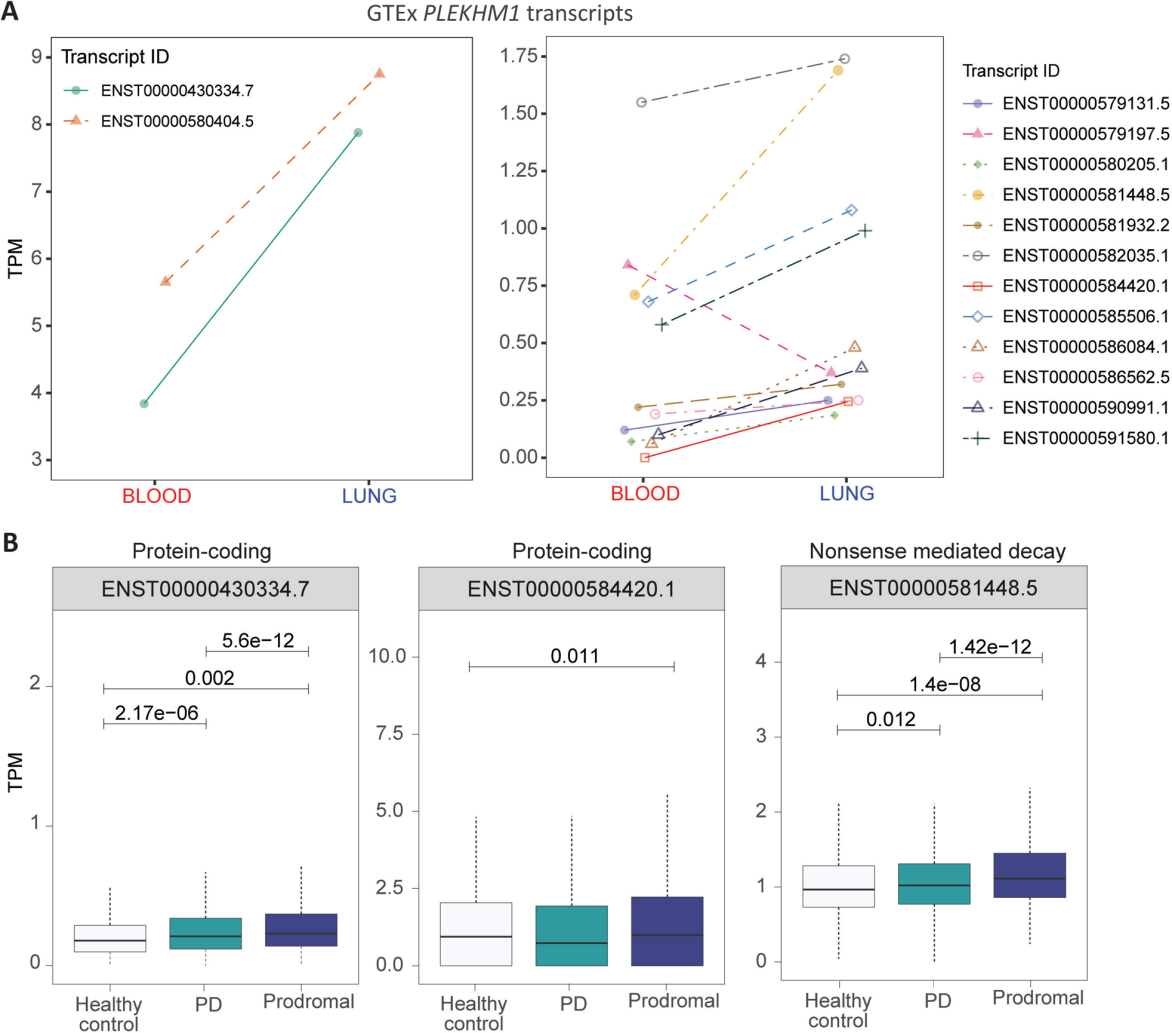
The opposing odds ratios for *PLEKHM1* in blood (OR = 30.24) and lung (OR = 0.45) may be explained by the observed tissue-specific differences in transcript expression. **A** *PLEKHM1* transcript expression (TPM) levels in blood and lung tissues in GTEx samples (data accessed on 06–12–2023). The *x*-axis labels are color-coded to indicate whether the gene is associated with increased risk (red) or protection (blue) against PD. Two separate graphs were plotted to visually differentiate smaller and larger y-axis scales. **B** *PLEKHM1* transcripts expression among healthy controls, PD patients, and prodromal individuals in the PPMI cohort. Significant differences in the expression of specific protein-coding and non-coding transcripts were observed in PD compared to healthy controls and prodromal individuals

*PLEKHM1* encodes a cytosolic adapter protein that coordinates endocytic trafficking and facilitates autophagosome-lysosome fusion [48]. *PLEKHMI* is transcribed into ≥8 protein-coding transcripts (Ensembl) that encode PLEKHM1 isoforms ranging in size from 113 AA to 1056 AA (https://www.proteinatlas.org/ENSG00000225190-PLEKHM1/summary/gene). The rs8070723-*PLEKHM1* pairing identified in whole blood increased risk (OR=30.2 ± 14.8, *p*=8.95 × 10^−21^) for PD, whilst the rs56168933-*PLEKHM1* pairing identified in the lung reduced PD risk (OR = 0.45 ± 0.37, *p*=5.92 × 10^−16^). As we observed for *SNCA*, the switch in risk or protection for PD associated with differences in regulation of *PLEKHM1* was potentially explained by altered expression levels of transcripts in whole blood and lung tissues. In GTEx, *PLEKHM1* transcripts showed distinct tissue-specific expression patterns between blood and lung. All protein-coding, non-coding, and nonsense-mediated decay (NMD) transcripts—except for ENST00000579197.5 (NMD)—were more highly expressed in lung (Fig 4A; Supplementary Table 14). Notably, the protein-coding transcript ENST00000584420.1 showed complete loss of expression in blood.

The regulatory sceQTLs of *PLEKHM1* identified in blood (rs8070723) and lung (rs56168933) are both located within the chromosome 17q21.31 inversion which has two haplotype H1 and H2). These two SNPs are separated by 568,746 bp and are not in linkage disequilibrium (*r*^2^<0.02)*.* rs8070723 is a H1 haplotype tag SNP [49], potentially linking the regulatory impact of the rs8070723 locus to the inversion status. However, the orientation of the 0.9Mb chr17q21.31 inversion (H1/H2) in individuals will not significantly alter the relative distance (in both linear sequence and 3-dimensional organization) between *PLEKHM1* and these two SNPs.

In the PPMI cohort, we further assessed transcript-level differences between healthy controls, PD patients, and prodromal individuals. Several *PLEKHM1* transcripts, including both protein-coding and NMD isoforms, were significantly upregulated in PD and prodromal individuals compared to healthy controls (Fig 4B, Supplementary Fig. 2). Additional *PLEKHM1* isoforms highlighted consistent overexpression in PD (Supplementary Fig. 2), reinforcing that tissue-specific protein-coding, non-coding, and NMD transcript expression differences may contribute to altered PD risk. However, unlike *SNCA*, the effects of these isoform changes are not yet understood, emphasising the need for more research to clarify the impact of variations in *PLEKHM1* isoform expression.

### An Additional Eight Causal Genes Exhibit Tissue-Dependent Switching from Risk to Protection

Two-sample MR also identified that *ELOVL7*, *HSD3B7*, *LINC02210*, *RP11-1072 A3.3*, *STX1B*, *PRSS53*, *STX4*, and *ZSWIM7* switch direction of causal effects across different tissues (Supplementary Figs. 3–7). Consistent with our earlier observations for *SNCA* and *PLEKHM1*, tissue-specific isoform level differences were associated with risk or protection for these genes*.* Notably, *PRSS53* encodes a serine protease 53 and is comprised of 13 exons that are transcribed into ≥ 3 transcript isoforms. PRSS53-553 AA includes the serine endopeptidase active site and is currently the only characterized functional protein produced from the gene (https://www.proteinatlas.org/ENSG00000151006-PRSS53/summary/gene). The transcript encoding PRSS53-553 AA (ENST00000280606) is undetectable in the aorta where the OR is 1.8 (Supplementary Fig. 5 C). Taken together, these findings highlight the importance of tissue-specific isoform expression in PD risk and protection.

## Discussion

In this study, we employed two-sample MR on tissue-specific gene regulatory networks to identify genes that are causally related to PD. We observed that these genes were often regulated by different sceQTLs in specific tissues. This is consistent with our understanding of the dynamics and combinatorial nature of gene regulation. Interestingly, we identified ten genes (*ELOVL7*, *HSD3B7*, *PLEKHM1*, *PRSS53*, *SNCA*, *STX1B*, *STX4*, *ZSWIM7*, *LINC02210*, and *RP11-1072 A3.3*) that were causally related with PD but associated sceQTLs that linked to both protection and risk for PD in a tissue-specific manner. Further investigation of tissue-specific expression profiles of the causal genes identified specific isoforms (e.g. α-Syn-112 AA) that are linked to PD. Consistent with this, synuclein aggregation data identified that PD patients with *SNCA* isoform expression altering genotypes were aggregation positive (as assessed by SAA). Given that genes, especially those expressed in neurons, undergo alternative splicing to express multiple protein isoforms with differing functions and sub-cellular locations, the identification of disease-associated isoforms provides greater insight into PD disease mechanisms. Notably, our analysis revealed that 14 of the PD causal genes identified, and specific isoforms, impacted the autophagosome-lysosome fusion pathway.

The two-sample MR approach we applied in this study incorporates tissue specific networks to enable the identification of putative PD causal sceQTL-gene pairs in adult tissues [19]. However, this does not mean that the transcriptional changes detected in a tissue are those responsible for the causal relationship between the sceQTL-gene pair and PD. Rather, the inherited germline variants that are identified are likely to have an effect throughout the life-time development of PD. As such, the fact that they are causally related to PD is more important than the tissue in which they were identified. This conclusion is based upon the fact that nuclear structure [50] and transcriptional profiles are developmental and tissue-specific [51, 52]. Thus, the tissues used in the assay represent a set of possible regulatory networks that capture different combinatorial effects of the allele-specific control elements on gene expression.

Our analysis revealed the genes that impact autophagosome-lysosome fusion in PD risk. Within cells, autophagosome-lysosome fusion is a critical process for the degradation of abnormal organelles and aggregation-prone proteins, which is essential for maintaining cellular health, as is the recycling of degraded components for re-use in the cell. Notably, our analysis revealed that a subset of causal genes, exhibiting both risk and protective effects for PD, is involved in autophagosome-lysosome fusion. Based on this finding, we hypothesise that life-time alterations in the isoforms transcribed from these causal genes contribute to PD risk by impairing/disrupting autophagosome–lysosome fusion. The connection between specific isoforms of PD causal genes and autophagosome-lysosome fusion highlights the importance of isoform-specific effects on cellular processes and PD risk, providing new and more detailed insights into PD pathogenesis.

Copy number variations of chr16p11.2 and inversions of chr17q21.31 are frequently linked to neurological and neurodevelopmental disorders [53–55]. Two sample MR indicated that these regions were enriched for putative PD causal genes. The genes that implicate chr16p11.2 and chr17q21.31 in PD pathogenesis were not all enriched within the autophagosome-lysosome fusion pathway. Notably, *KAT8* and *KANSL1—*essential components of the chromatin-modulating non-specific lethal (NSL) complex [56, 57]—are localized within these cytobands. The NSL complex likely acts as a master regulator of multiple disease-related pathways, as *KANSL1* and *KAT8* knockdown significantly affects the expression of PD-associated genes, including *PINK1* and *DGKQ* [56]. We contend that copy number variation of chr16p11.2 and inversion of chr17q21.31 likely results in fewer or extra copies of genes (chr16p11.2) and the disruption of transcriptional regulation of the genes within these regions (chr17q21.31) [58]. Future research should use CRISPR base editing to test how the identified variants affect cell lines with these regions in different orientations and copy numbers. Confirming that disruptions in the regulation of genes within chr16p11.2 and chr17q21.31 impact the NSL complex and PD-associated genes could lead to new treatments for about 80% of PD patients with these structural predispositions.

Gene switches between risk and protection for PD provide crucial insights into understanding PD risk. It is the differences in the gene transcript isoform(s) expression pattern (i.e. the proportions of isoforms for each gene), and not overall gene expression levels, that appears to drive tissue-specific risk or protection status. Therefore, comparing transcript expression patterns across the tissues identified the isoforms responsible for the switch in PD risk. For all the risk-switching genes, the proportions that particular isoforms contributed to the total expression of the causal genes differed between the tissues where the risk and protective effect was observed. For example, the α-Syn-66 AA was not detected in the skin (SKIN_NEX; median TPM = 0), while the α-Syn-112 AA encoding transcript accounts for a greater proportion of the α-Syn transcripts in skin (SKIN_NEX; median TPM = 0.39), a tissue increasingly recognised for its relevance in PD pathology [29], than in the blood (BLOOD; median TPM = 0.21). While our analysis supports the notion that alternate transcript usage influences tissue-specific PD risk [59, 60], the relationship between transcript isoform patterns and disease risk remains complex. Transcript isoforms for *SNCA* and other genes have been implicated in PD pathogenesis before [61, 62]. For example, real-time PCR studies have identified alternative splice isoforms of *GDNF* and *SRRM2* genes in PD patient samples [63, 64]. Yet whether there are causal relationships between different gene isoforms and PD remains unclear. Thus there is a need for functional assays to define the biological significance of these splice variants and if they make a causal contribution to PD pathogenesis.

Given that misfolded α-Syn is a hallmark of PD and is implicated in disease progression through templating and aggregation mechanisms, the observed isoform shifts may likely have functional implications. Studies have shown that misfolded α-Syn species contribute to Lewy body pathology in PD patient brains and can propagate through prion-like mechanisms, spreading to interconnected brain regions [59]. These findings align with previous reports indicating that specific α-Syn isoforms have differential aggregation propensities and may differentially contribute to neurotoxicity [65]. The increased proportions of α-Syn-112 AA and α-Syn-115 AA in risk-associated tissue suggests that these isoforms may be particularly prone to misfolding and seeding activity, making them potential molecular markers for early PD detection. Future studies validating these isoforms as biomarkers in peripheral tissues could facilitate early, non-invasive detection of PD and improve our understanding of isoform-specific contributions to PD pathology.

The association of risk and protection status with specific variants offers valuable insights for population-based stratification and targeted treatment of different symptoms of PD. For instance, the allele-specific variants surrounding the *PLEKHM1* gene, rs8070723 and rs56168933, modulate tissue-specific expression of *PLEKHM1*. Notably, rs8070723 is linked to the H1 haplotype of the 17q.21.31 locus, previously implicated in PD risk [49]. Thus, integrating knowledge of allele-specific control mechanisms with isoform-level expression data enhances our ability to pinpoint the precise molecular mechanisms of PD. Future characterisation of these isoforms will enable better stratification of individuals, based on their genetic risk factors, and the development of targeted therapeutic approaches specific to PD subtypes. This refined understanding of genetic architecture of PD will improve treatment outcomes for PD patients as even transcriptional isoforms encoding the same protein isoforms but with different 5′ and 3′ UTRs are likely to have different RNA half-lives (miRNA binding to 3′ UTR), translational efficiency, sub cellular localisations for localised translation etc…

The aggregation of αSyn is a hallmark of PD pathogenesis, with autophagy playing a crucial role in the clearance of these aggregates. When autophagy is compromised, it leads to the accumulation of α-syn aggregates, worsening neurodegeneration [66]. *PLEKHM1*, *SNCA*, and *LRRK2* are enriched in autophagy-related pathways (Table 1), underscoring the potential importance of autophagy in PD pathogenesis. Specifically, the interaction of *PLEKHM1* with the HOPS-SNARE complexes facilitates autophagosome-lysosome fusion. The depletion of *PLEKHM1* impairs autophagy-mediated degradation [67], potentially contributing to the pathological accumulation of aggregates. Similarly, the pathogenic A30P mutation in α-synuclein creates an imbalance between α-synuclein production and degradation, leading to its accumulation and inhibition of autophagy [68]. Observations support that chronic, endogenous accumulation of αSyn impairs autophagosome-lysosome fusion process by inhibiting the activity of the SNARE protein ykt6, thereby disrupting the fusion [69], which underscores the importance of understanding the interplay between αSyn pathology and cellular degradation mechanisms in PD. The results of our study support these earlier observations as we identified links between common genetic variants and genes (*PLEKHM1*, *SNCA*, etc.) that are essential for autophagic clearance mechanisms, revealing the regulatory networks driving PD pathogenesis.

The potential contribution of autophagosome-lysosome fusion to PD is multifaceted and it represents a possible environmental interface. Head trauma and traumatic brain injury, both risk factors for PD, impair the flux through the macroautophagy pathway and can promote neurodegeneration [17, 70, 71]. For example, Ding *et al*. identified that the protein DNALI1 promotes neurodegeneration after traumatic brain injury by inhibiting autophagosome-lysosome fusion [71]. Furthermore, evidence for environmental contaminants (e.g. [72, 73]) impacting autophagosome-lysosome fusion includes the 70% increased risk of PD development in Camp Lejeune veterans who served there between 1975 and 1985 when the water was contaminated with trichloroethylene [74], which impairs autophagy [75]. Genetic variation impacting genes involved in autophagosome-lysosome fusion and αSyn pathology may help explain individual susceptibility to environmental impacts and thus risk of developing PD.

MR has been used previously to investigate gene expression and its causal relationship with PD risk [15, 19]. While our novel approach has uncovered critical pathogenic mechanisms by focussing on tissue-specific isoform expression networks associated with PD progression [19], several points merit further clarification. First, although our data indicate that transcripts encoding α-Syn-112 AA and α-Syn-66 AA are elevated in prodromal individuals relative to both healthy controls and PD patients, the absence of a clear difference for α-Syn-112 AA between healthy and PD groups raises the possibility that this observation may, in part, reflect sample selection bias within the prodromal cohort. It remains to be determined whether the elevated isoform expression represents an early pathogenic event in PD progression or an artifact of cohort stratification. Longitudinal studies and independent replication cohorts will be essential to resolve this ambiguity.

Second, while the two-sample MR analysis robustly suggests that tissue-specific isoform proportions contribute to PD risk—particularly through mechanisms such as impaired autophagosome–lysosome fusion—the causal role of these isoform shifts remains inferential. MR provides a valuable framework for suggesting causality; however, direct experimental validation is required to confirm that alterations in isoform expression indeed drive the observed cellular and pathological changes. Future studies using MR to dissect isoform-level regulation in disease-relevant tissues, such as the substantia nigra or peripheral tissues implicated in PD pathogenesis, could provide valuable insights into the mechanisms underlying isoform-specific contributions to PD susceptibility and disease progression. Such approaches may help identify key regulatory elements and pathways driving α-Syn pathology, ultimately informing potential therapeutic targets and biomarker development. In addition, the inclusion of allele-specific transcript isoform data derived from long-read transcriptome sequencing into future studies would enable the precise identification of the pathogenic isoforms of the gene. This would offer a more accurate and detailed understanding of the isoform-specific regulatory mechanisms contributing to PD.

In summary, integrating tissue-specific gene regulatory networks with two-sample MR identified causal genes and their common regulatory elements that modulate PD risk. Our findings highlight the critical role of isoform-specific expression in influencing disease risk and outcomes, particularly in autophagosome-lysosome fusion. Our study challenges conventional gene-centric approaches, advocating for a revised framework that accounts for gene isoforms within GWAS interpretations, and highlights the potential isoform-based therapies as a valuable tool for potential therapeutics in readily identifiable patients. While our integrated approach provides novel insights into isoform-specific contributions to PD risk, further experimental and longitudinal investigations are required to confirm the proposed mechanisms and fully understand the interplay between genetic variation, isoform expression, and disease pathology.

## Supplementary Information

Below is the link to the electronic supplementary material.

Supplementary file1 (PDF 1371 KB)

Supplementary file2 (PDF 774 KB)

## Data Availability

All data analysed or produced throughout this research is detailed in the article or Supplementary Material. Additionally, publicly accessible datasets obtained and utilised in our study were Genotype-Tissue Expression (GTEx) Portal (https://www.gtexportal.org/home/). Custom scripts used for data analyses and visualisation are available at: https://github.com/Genome3d/PD_multi_tissue_MR.git. Code for bioinformatics software or algorithm is available at: CoDeS3D (https://github.com/Genome3d/codes3d-v1.git), Multimorbid3D (https://github.com/Genome3d/multimorbid3D.git), TwoSampleMR (https://github.com/MRCIEU/TwoSampleMR.git).
