# Supplementary material for: Inherited Genetic Variation in Parkinson’s Disease: Convergence on Impaired Autophagosome-Lysosome Fusion Through the Altered Expression of mRNA Isoforms
Source: Mol Neurobiol. 2025 Jun 2;62(10):12824–38. doi: 10.1007/s12035-025-05101-2 (PMC12433354; doi:10.1007/s12035-025-05101-2)
Supplement: Supplementary file 1 — Supplementary file1 (PDF 1371 KB) [file 12035_2025_5101_MOESM1_ESM.pdf]

Inherited genetic variation in Parkinson's disease: convergence on impaired autophagosome-lysosome fusion through the altered expression of mRNA isoforms

S. Gokuladhas[1], C. Miller[1], A. Cooper[2,3], J.M. O'Sullivan[1,2,4,5,6*]

1.The Liggins Institute, University of Auckland, Auckland 1023, New Zealand.
2. Australian Parkinson's Mission, Garvan Institute of Medical Research, Sydney, New South Wales, Australia
3. School of Clinical Medicine UNSW Sydney, Sydney, NSW, Australia.
4. Maurice Wilkins Centre for Molecular Biodiscovery, Auckland 1010, New Zealand.
5. MRC Lifecourse Epidemiology Unit, University of Southampton, United Kingdom.
6. Singapore Institute for Clinical Sciences, Agency for Science, Technology and Research (A*STAR), Singapore, Singapore.

*Correspondence to: Justin M. O'Sullivan
Full address justin.osullivan@auckland.ac.nz

Supplementary Figures

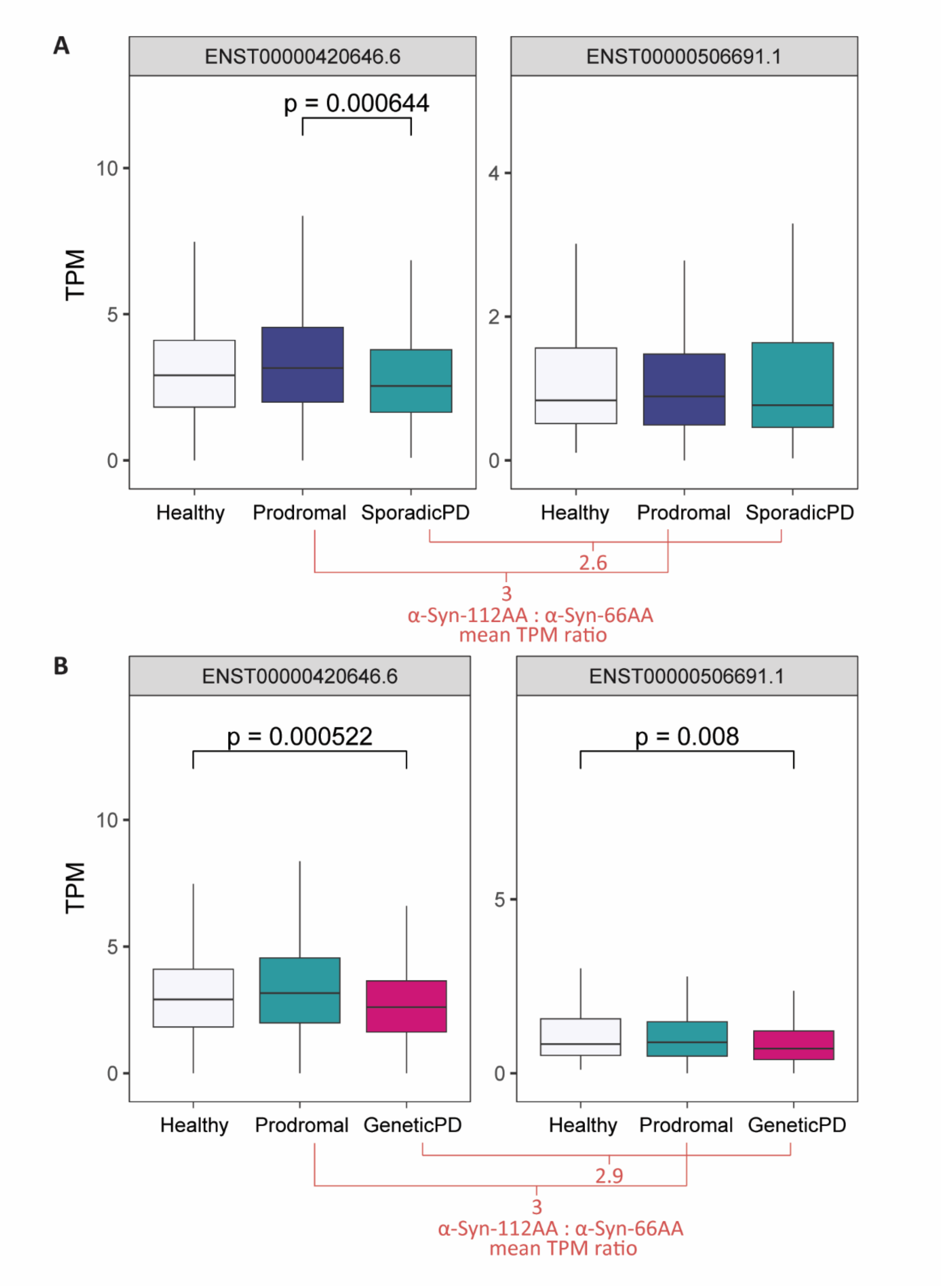

Supplementary Fig 1. A consistent change in SNCA transcript ratios was observed between prodromal and PD after subgroup stratification. Comparison of transcript expression levels for the α-Syn-112AA (ENST00000420646.6) and α-Syn-66AA (ENST00000506691.1) isoforms identified a minor change in the ratio between both forms of PD (sporadic and genetic) and prodromal participants within the PPMI cohort.

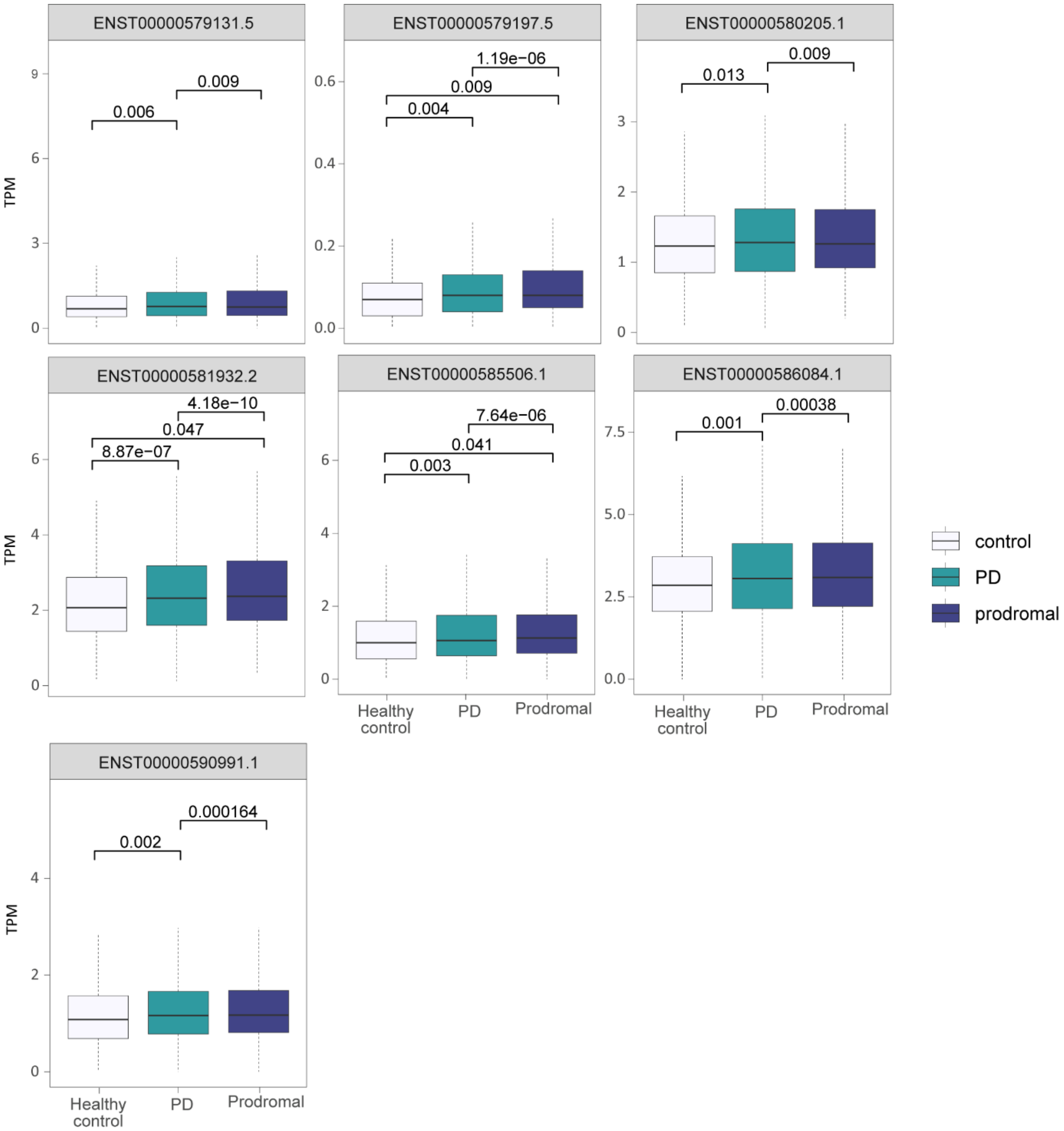

Supplementary Fig 2. *PLEKHM1* transcripts expression among healthy controls, PD patients, and prodromal individuals in the PPMI cohort. Significant differences in the expression of specific protein-coding and non-coding transcripts were observed in PD compared to healthy controls and prodromal individuals.

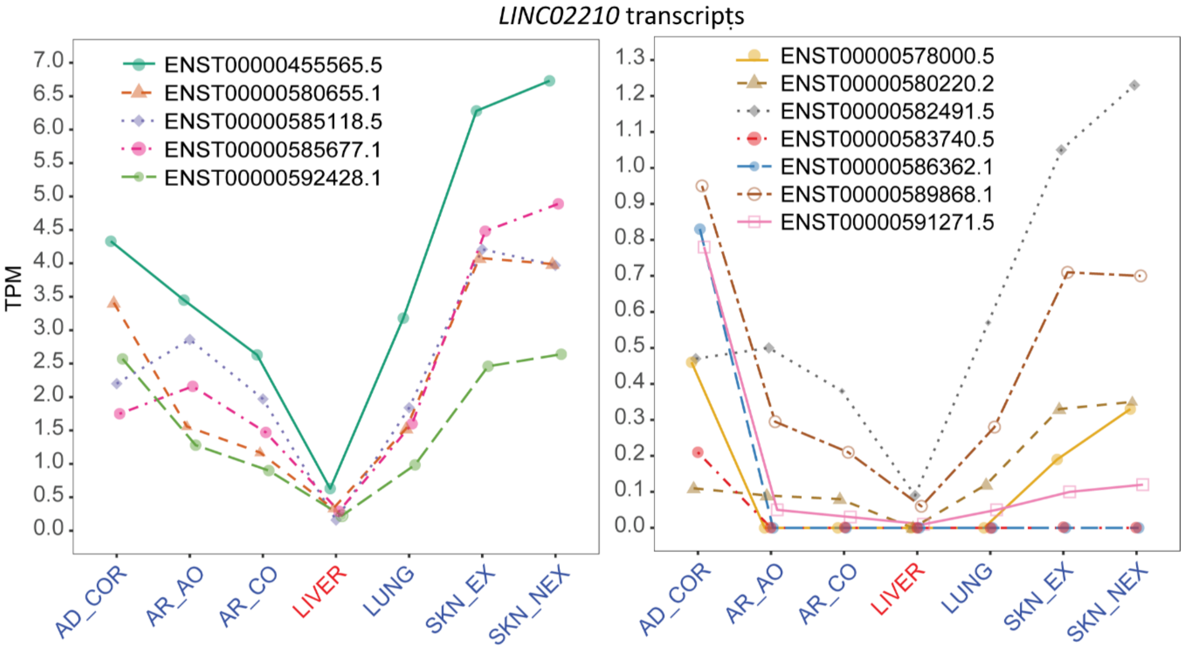

Supplementary Fig 3. MR estimated causal effects may be attributed to variations in transcript level expression of transcript isoforms across tissues. Transcript isoform level expression changes quantified in GTEx cohort for *LINC02210* transcripts**.** The x-axis labels are color-coded to indicate whether the gene is associated with increased risk (red) or protection (blue) against PD. Two separate graphs were plotted to visually differentiate smaller and larger y-axis scales.

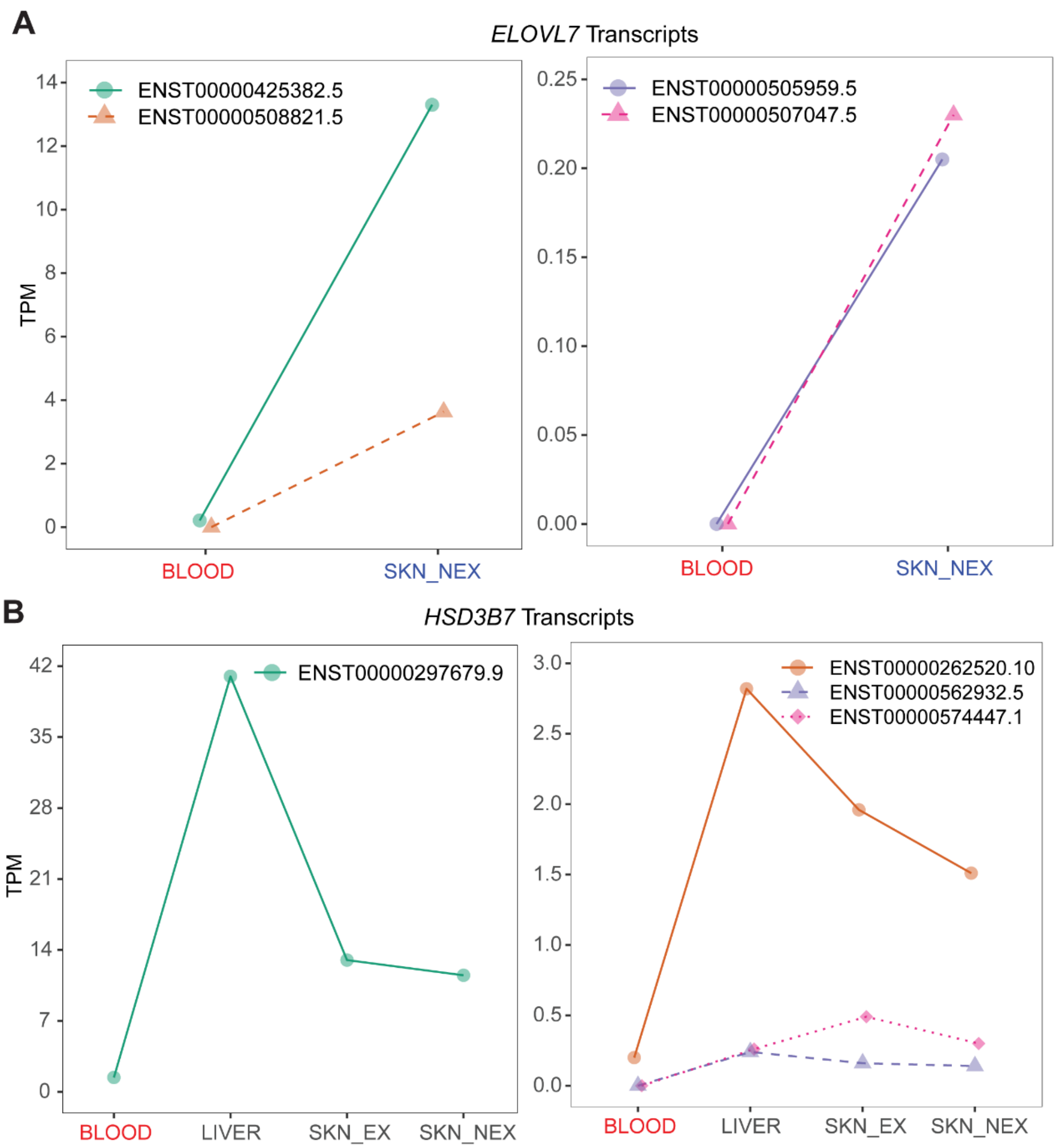

Supplementary Fig 4. MR estimated causal effects may be attributed to variations in transcript level expression of transcript isoforms across tissues. Transcript isoform level expression changes quantified in GTEx cohort for **A.** *ELOVL7* and **B.** *HSD3B*7**.** The x-axis labels are color-coded to indicate whether the gene is associated with increased risk (red) or protection (blue) against PD.

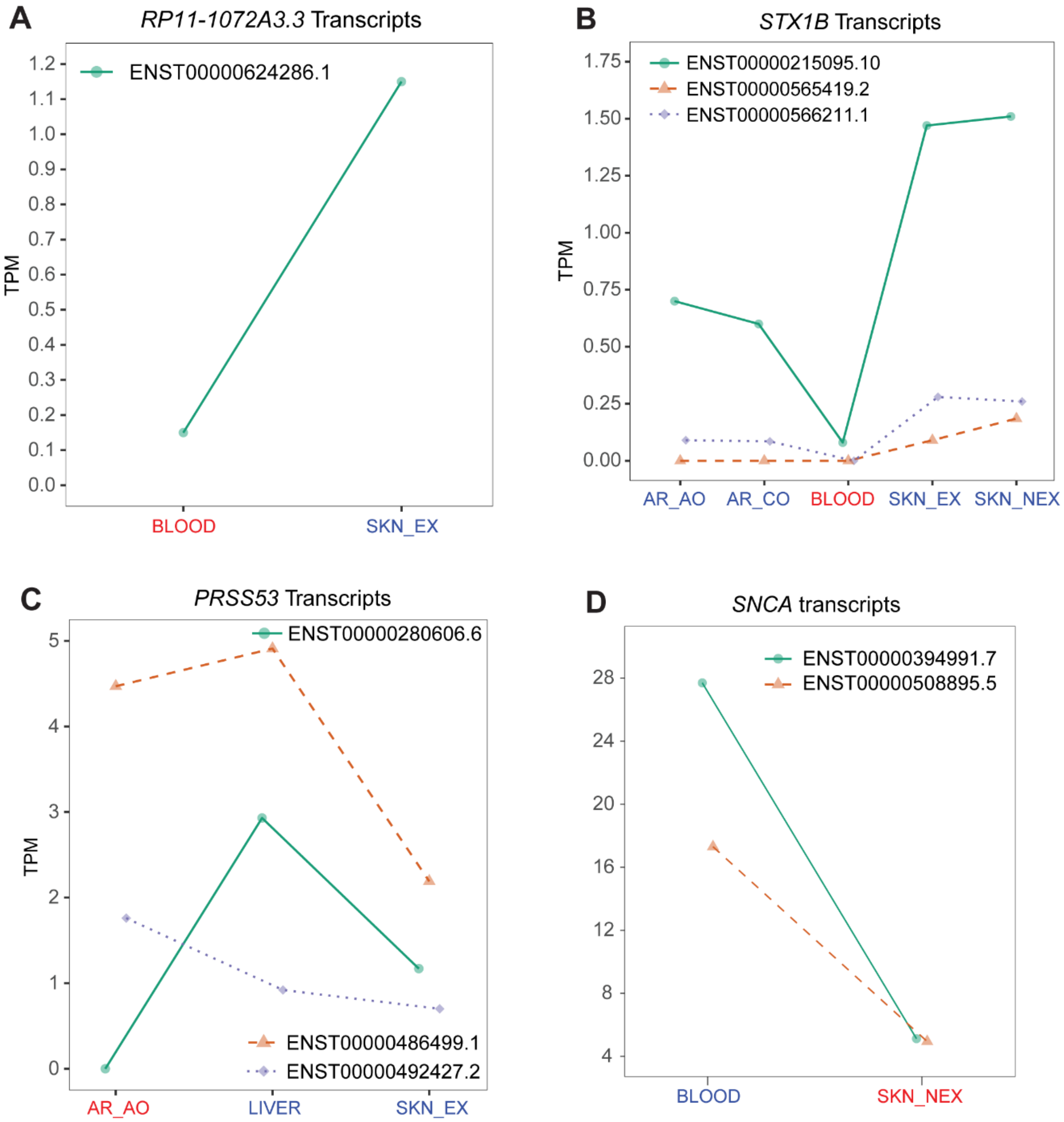

Supplementary Fig 5. MR estimated causal effects may be attributed to variations in transcript level expression of genes isoforms across tissues. Transcript isoform level expression changes quantified in GTEx cohort for **A.** *RP11-1072A3.3*, **B.** *STX1B*, **C.** *PRSS53*, and **D.** *SNCA* transcripts**.** The x-axis labels are color-coded to indicate whether the gene is associated with increased risk (red) or protection (blue) against PD.

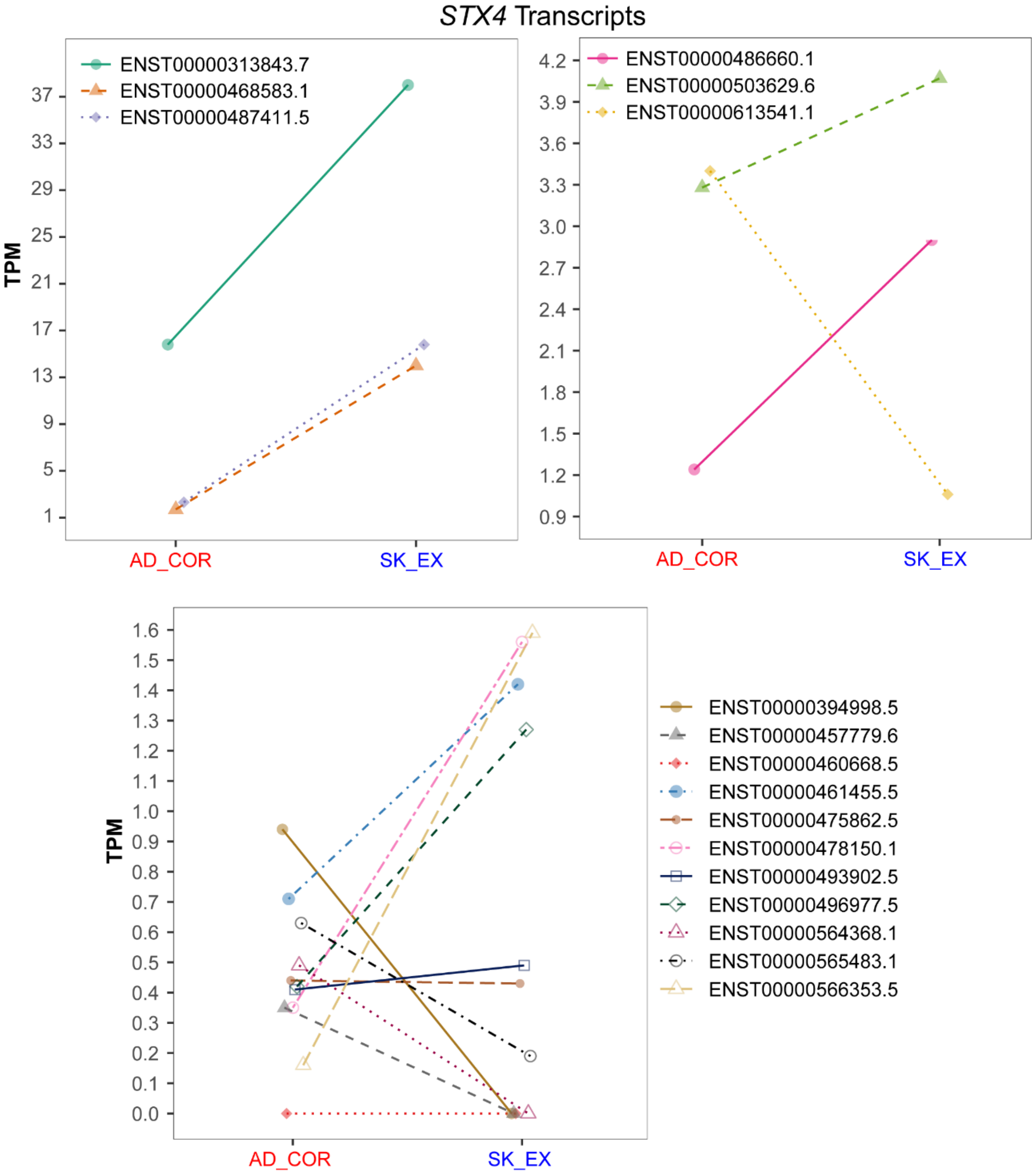

Supplementary Fig 6. MR estimated causal effects may be attributed to variations in transcript level expression of transcript isoforms across tissues. Transcript isoform level expression changes quantified in GTEx cohort for *STX4* transcripts. The x-axis labels are color-coded to indicate whether the gene is associated with increased risk (red) or protection (blue) against PD.

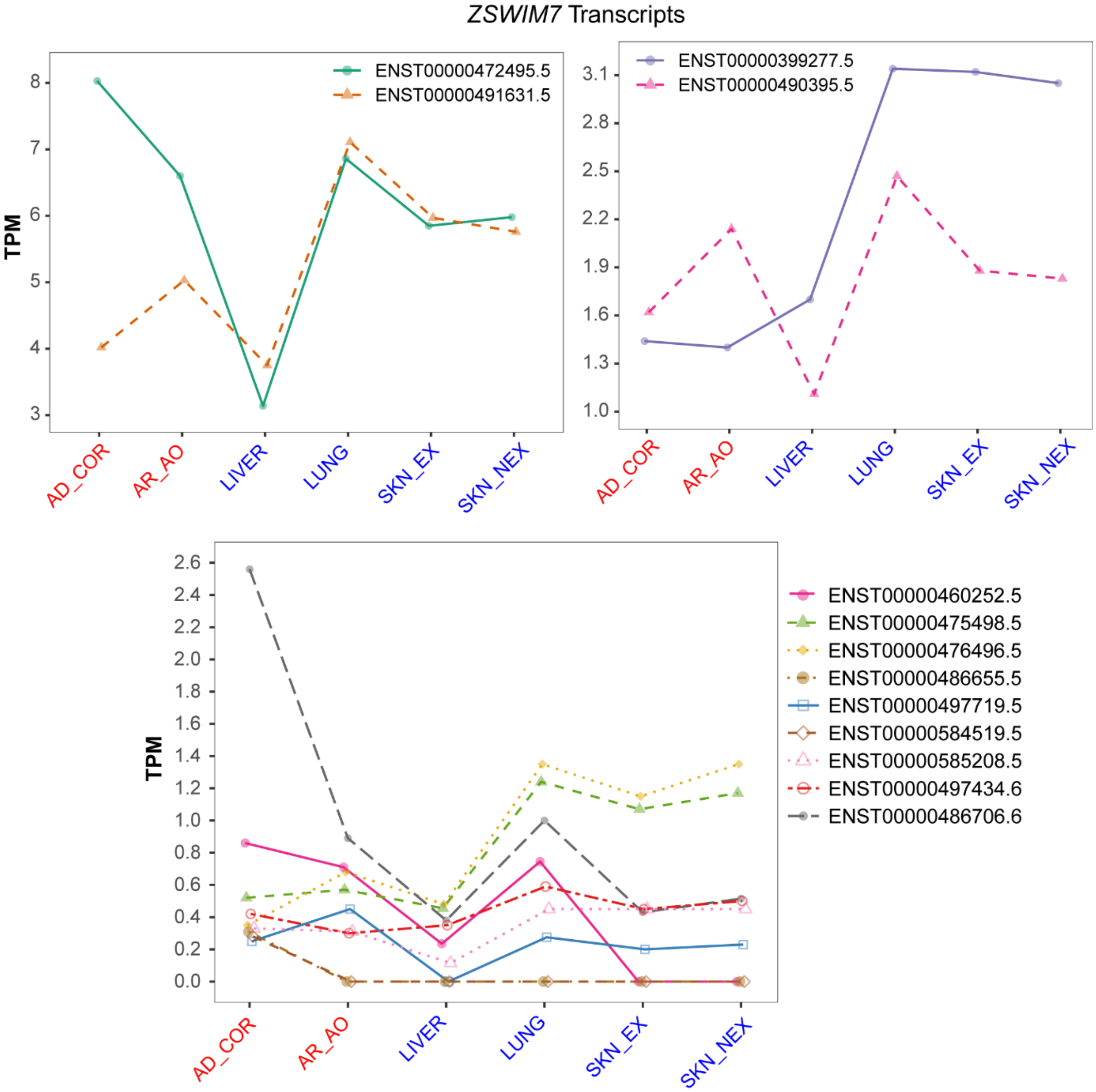

Supplementary Fig 7. MR estimated causal effects may be attributed to variations in transcript level expression of transcript isoforms across tissues. Transcript isoform level expression changes quantified in GTEx cohort for *ZSWIM7* transcripts. The x-axis labels are color-coded to indicate whether the gene is associated with increased risk (red) or protection (blue) against PD.
