# Supplementary material for: Inherited Genetic Variation in Parkinson’s Disease: Convergence on Impaired Autophagosome-Lysosome Fusion Through the Altered Expression of mRNA Isoforms
Source: Mol Neurobiol. 2025 Jun 2;62(10):12824–38. doi: 10.1007/s12035-025-05101-2 (PMC12433354; doi:10.1007/s12035-025-05101-2)
Supplement: Supplementary file 2 — Supplementary file2 (PDF 774 KB) [file 12035_2025_5101_MOESM2_ESM.pdf]

**Supplementary Table 1. Hi-C and eQTL datasets used for generating tissues-specific gene regulatory networks (GRNs)**

| Tissue | Hi-C cell lines | Hi-C data source | eQTL tissue source | sceQTLs (#) | Genes (#) | sceQTLg-gene interactions (#) |
|---|---|---|---|---|---|---|
| AD_COR | Dorsolateral prefrontal cortex cells | https://pubmed.ncbi.nlm.nih.gov/27851967/ | GTEx | 862963 | 14427 | 1050107 |
| BLOOD | Peripheral blood B cells | https://pubmed.ncbi.nlm.nih.gov/29883495/ | GTEx | 1077379 | 14862 | 1713829 |
| | Peripheral blood CD4+ T cells | | | | | |
| | Peripheral blood CD8+ T cells | | | | | |
| | Peripheral blood T cells | https://pubmed.ncbi.nlm.nih.gov/32203470/ | | | | |
| LIVER | HepG2 | https://www.ncbi.nlm.nih.gov/pubmed/22955616 | GTEx | 327210 | 8097 | 468759 |
| | LIVER cells | https://pubmed.ncbi.nlm.nih.gov/27851967/ | | | | |
| | Endothelial of hepatic sinusoid cells | https://www.ncbi.nlm.nih.gov/pubmed/22955616 | | | | |
| LUNG | LUNG cells | https://pubmed.ncbi.nlm.nih.gov/27851967/ | GTEx | 740028 | 15855 | 872830 |
| AR_AO | Coronary artery smooth muscle cells | https://pubmed.ncbi.nlm.nih.gov/32513244/ | GTEx | 875979 | 14748 | 1342727 |
| | Left ventricle cells | https://pubmed.ncbi.nlm.nih.gov/27851967/ | | | | |
| | Aorta cells | | | | | |
| | TeloHAEC Lalonde2019 | https://pubmed.ncbi.nlm.nih.gov/31287004 | | | | |
| AR_CO | Coronary artery smooth muscle cells | https://pubmed.ncbi.nlm.nih.gov/32513244/ | GTEx | 374761 | 9115 | 489811 |
| | Left ventricle cells | https://pubmed.ncbi.nlm.nih.gov/27851967/ | | | | |
| | Aorta cells | | | | | |
| | TeloHAEC Lalonde2019 | https://pubmed.ncbi.nlm.nih.gov/31287004 | | | | |
| SKN_EX | NHEK | https://www.ncbi.nlm.nih.gov/pubmed/25497547 | GTEx | 1208794 | 18529 | 2054680 |
| | RPMI7951 | https://pubmed.ncbi.nlm.nih.gov/22955616/ | | | | |
| | SK-MEL-5 | | | | | |
| SKN_NEX | NHEK | https://www.ncbi.nlm.nih.gov/pubmed/25497547 | GTEx | 1076235 | 17614 | 1752173 |
| | RPMI7951 | https://pubmed.ncbi.nlm.nih.gov/22955616/ | | | | |
| | SK-MEL-5 | | | | | |

| Supplementary Table | Title | DOI |
|---|---|---|
| 2 | AD_COR gene regulatory network | https://doi.org/10.17608/k6.auckland.27085558 |
| 3 | BLOOD gene regulatory network | https://doi.org/10.17608/k6.auckland.27106225 |
| 4 | AR_AO gene regulatory network | https://doi.org/10.17608/k6.auckland.27106282 |
| 5 | AR_CO gene regulatory network | https://doi.org/10.17608/k6.auckland.27106288 |
| 6 | LUNG gene regulatory network | https://doi.org/10.17608/k6.auckland.27106291 |
| 7 | LIVER gene regulatory network | https://doi.org/10.17608/k6.auckland.27106297 |
| 8 | SKN_EX gene regulatory network | https://doi.org/10.17608/k6.auckland.27106303 |
| 9 | SKN_NEX gene regulatory network | https://doi.org/10.17608/k6.auckland.27106309 |

**Supplementary Table 10. The 79 putative causal genes for Parkinson's disease (PD) were identified across eight tissues using two-sample Mendelian randomization analysis**

| exposure | outcome | samplesize | SNP | beta | standard error | p-value | odds ratio (OR) | OR_lci95 | OR_uci95 | tissue |
|---|---|---|---|---|---|---|---|---|---|---|
| AC005082.12 | PD | 482730 | rs7808488 | -0.24754584 | 0.042333926 | 4.99E-09 | 0.780714432 | 0.71854975 | 0.84825724 | AR_AO |
| AC005082.12 | PD | 482730 | rs858306 | -0.205173444 | 0.035326556 | 6.33E-09 | 0.814506033 | 0.76001775 | 0.87290077 | SKN_NEX |
| AC005082.12 | PD | 482730 | rs6461687 | -0.269234751 | 0.048537341 | 2.91E-08 | 0.763963893 | 0.69463556 | 0.84021156 | LIVER |
| AC005082.12 | PD | 482730 | rs858295 | -0.242128911 | 0.041015099 | 3.56E-09 | 0.784954982 | 0.72432253 | 0.85066293 | LUNG |
| AC005082.12 | PD | 482730 | rs858306 | -0.205173444 | 0.035326556 | 6.33E-09 | 0.814506033 | 0.76001775 | 0.87290077 | SKN_NEX |
| AC079630.4 | PD | 482730 | rs2708418 | -0.910076557 | 0.196981504 | 3.84E-06 | 0.402493409 | 0.27358024 | 0.59215147 | BLOOD |
| ADORA2B | PD | 482730 | rs4792713 | 0.471333376 | 0.094155249 | 5.56E-07 | 1.60212901 | 1.33214322 | 1.92683289 | AD_COR |
| ADORA2B | PD | 482730 | rs1045599 | 0.118229586 | 0.023111546 | 3.13E-07 | 1.125502481 | 1.07565627 | 1.17765858 | AR_AO |
| ADORA2B | PD | 482730 | rs1045599 | 0.1339039 | 0.026175565 | 3.13E-07 | 1.143282945 | 1.08610705 | 1.20346874 | AR_CO |
| ADORA2B | PD | 482730 | rs8066902 | 0.376407072 | 0.071517344 | 1.42E-07 | 1.457040134 | 1.26646946 | 1.67628673 | SKN_EX |
| ADORA2B | PD | 482730 | rs2285583 | 0.380572319 | 0.07438459 | 3.12E-07 | 1.463121723 | 1.26462865 | 1.69276979 | SKN_NEX |
| ADORA2B | PD | 482730 | rs12937434 | 0.35959642 | 0.067017437 | 8.06E-08 | 1.432751069 | 1.25638964 | 1.63386863 | BLOOD |
| ADORA2B | PD | 482730 | rs1045599 | 0.127464292 | 0.024916749 | 3.13E-07 | 1.135944305 | 1.08180124 | 1.19279718 | LIVER |
| ADORA2B | PD | 482730 | rs2779210 | 0.368613476 | 0.072228316 | 3.34E-07 | 1.445728686 | 1.25488756 | 1.6655926 | LUNG |

| exposure | outcome | samplesize | SNP | beta | standard error | p-value | odds ratio (OR) | OR_lci95 | OR_uci95 | tissue |
|---|---|---|---|---|---|---|---|---|---|---|
| ADORA2B | PD | 482730 | rs2285583 | 0.380572319 | 0.07438459 | 3.12E-07 | 1.463121723 | 1.26462865 | 1.69276979 | SKN_NEX |
| AK3P3 | PD | 482730 | rs73272053 | 0.325301613 | 0.067095814 | 1.25E-06 | 1.38444815 | 1.21384598 | 1.5790279 | SKN_NEX |
| AK3P3 | PD | 482730 | rs73272053 | 0.325301613 | 0.067095814 | 1.25E-06 | 1.38444815 | 1.21384598 | 1.5790279 | SKN_NEX |
| ARHGAP27 | PD | 480593 | rs79724577 | -0.611146559 | 0.075470447 | 5.59E-16 | 0.542728243 | 0.4681022 | 0.62925136 | AD_COR |
| ARHGAP27 | PD | 480593 | rs62065396 | -0.44667869 | 0.054550141 | 2.65E-16 | 0.639749433 | 0.57487827 | 0.71194087 | SKN_EX |
| ARHGAP27 | PD | 480593 | rs7209501 | -0.488833881 | 0.06019797 | 4.65E-16 | 0.613341206 | 0.54508044 | 0.69015031 | SKN_NEX |
| ARHGAP27 | PD | 480593 | rs7209501 | -0.488833881 | 0.06019797 | 4.65E-16 | 0.613341206 | 0.54508044 | 0.69015031 | SKN_NEX |
| ARL17A | PD | 480593 | rs241021 | -0.891417825 | 0.095319926 | 8.61E-21 | 0.410073927 | 0.34019208 | 0.49431082 | BLOOD |
| ARL17B | PD | 482730 | rs11656151 | 0.148468258 | 0.030959553 | 1.62E-06 | 1.160055975 | 1.09175614 | 1.23262863 | BLOOD |
| BACE2 | PD | 468692 | rs235925 | -0.314721911 | 0.101290296 | 0.001889146 | 0.729991849 | 0.5985466 | 0.89030344 | SKN_NEX |
| BACE2 | PD | 468692 | rs2776344 | -0.314721911 | 0.101290296 | 0.001889146 | 0.729991849 | 0.5985466 | 0.89030344 | SKN_NEX |
| BACE2 | PD | 468692 | rs3787938 | -0.314721911 | 0.101290296 | 0.001889146 | 0.729991849 | 0.5985466 | 0.89030344 | SKN_NEX |
| BCKDK | PD | 482730 | rs11862962 | 1.165368599 | 0.206992646 | 1.80E-08 | 3.207104799 | 2.13755577 | 4.8118142 | SKN_EX |
| BCKDK | PD | 482730 | rs749671 | 0.733962764 | 0.151765665 | 1.32E-06 | 2.083319976 | 1.54728543 | 2.8050559 | BLOOD |
| BST1 | PD | 482730 | rs4698119 | -1.061126163 | 0.1798184 | 3.61E-09 | 0.346065864 | 0.24327319 | 0.49229256 | BLOOD |
| CCDC158 | PD | 482730 | rs34078673 | -0.170791655 | 0.036866921 | 3.61E-06 | 0.842997189 | 0.78423166 | 0.90616625 | LUNG |
| CD38 | PD | 482730 | rs4698413 | -0.32032895 | 0.043364435 | 1.50E-13 | 0.725910209 | 0.66676125 | 0.79030632 | AD_COR |
| CTB-39G8.3 | PD | 480593 | rs439558 | -1.046649183 | 0.11165894 | 7.01E-21 | 0.351112293 | 0.28209801 | 0.43701067 | SKN_NEX |
| CTB-39G8.3 | PD | 480593 | rs439558 | -1.046649183 | 0.11165894 | 7.01E-21 | 0.351112293 | 0.28209801 | 0.43701067 | SKN_NEX |
| DGKQ | PD | 482730 | rs12510382 | -0.214965461 | 0.040277739 | 9.45E-08 | 0.806569298 | 0.7453437 | 0.87282422 | AR_AO |
| DGKQ | PD | 482730 | rs4690196 | -0.246673812 | 0.042973686 | 9.46E-09 | 0.781395533 | 0.71827539 | 0.85006251 | AR_CO |
| DGKQ | PD | 482730 | rs4690196 | -0.420583384 | 0.073270924 | 9.46E-09 | 0.656663621 | 0.5688182 | 0.75807544 | SKN_EX |
| DGKQ | PD | 482730 | rs4690196 | -0.436978685 | 0.076127192 | 9.46E-09 | 0.645985201 | 0.55644442 | 0.74993452 | SKN_NEX |
| DGKQ | PD | 482730 | rs4690196 | -0.436978685 | 0.076127192 | 9.46E-09 | 0.645985201 | 0.55644442 | 0.74993452 | SKN_NEX |

| exposure | outcome | samplesize | SNP | beta | standard error | p-value | odds ratio (OR) | OR_lci95 | OR_uci95 | tissue |
|---|---|---|---|---|---|---|---|---|---|---|
| DND1P1 | PD | 13839 | rs440778 | -0.188446902 | 0.029887646 | 2.88E-10 | 0.82824448 | 0.78111985 | 0.87821212 | AR_AO |
| DND1P1 | PD | 13839 | rs4485406 | -0.181418767 | 0.028772985 | 2.88E-10 | 0.834085998 | 0.78834945 | 0.88247597 | AR_CO |
| DND1P1 | PD | 482730 | rs55709241 | -0.183215794 | 0.019906275 | 3.45E-20 | 0.832588469 | 0.8007295 | 0.86571503 | SKN_EX |
| DND1P1 | PD | 482730 | rs62063777 | -0.188349863 | 0.020183192 | 1.04E-20 | 0.828324856 | 0.79619678 | 0.86174937 | SKN_NEX |
| DND1P1 | PD | 480593 | rs55974014 | -0.19369823 | 0.020718403 | 8.84E-21 | 0.823906497 | 0.79111946 | 0.85805236 | LIVER |
| DND1P1 | PD | 482730 | rs62063777 | -0.188349863 | 0.020183192 | 1.04E-20 | 0.828324856 | 0.79619678 | 0.86174937 | SKN_NEX |
| ELOVL7 | PD | 482730 | rs158918 | -0.972169648 | 0.144025133 | 1.48E-11 | 0.378261453 | 0.28523014 | 0.50163607 | SKN_NEX |
| ELOVL7 | PD | 480593 | rs34435485 | 0.787266122 | 0.139498032 | 1.67E-08 | 2.197380837 | 1.67171489 | 2.88834093 | BLOOD |
| ELOVL7 | PD | 482730 | rs158918 | -0.972169648 | 0.144025133 | 1.48E-11 | 0.378261453 | 0.28523014 | 0.50163607 | SKN_NEX |
| ERCC8 | PD | 482730 | rs10805374 | 0.58683591 | 0.12068769 | 1.16E-06 | 1.798289455 | 1.41947635 | 2.27819573 | LUNG |
| FAM200B | PD | 482730 | rs6839654 | 0.220232707 | 0.050209869 | 1.15E-05 | 1.246366735 | 1.1295523 | 1.37526171 | AD_COR |
| FAM200B | PD | 482730 | rs4301111 | 0.417894617 | 0.0927603 | 6.63E-06 | 1.518760614 | 1.26628122 | 1.821581 | AR_AO |
| FAM47E | PD | 482730 | rs7699714 | -0.168389551 | 0.036653295 | 4.35E-06 | 0.84502459 | 0.78644695 | 0.90796532 | AR_AO |
| FAM47E | PD | 482730 | rs7699714 | -0.165671317 | 0.036061618 | 4.35E-06 | 0.84732469 | 0.78950265 | 0.90938153 | AR_CO |
| FAM47E | PD | 482730 | rs7699714 | -0.155645947 | 0.0338794 | 4.35E-06 | 0.855862157 | 0.80087566 | 0.91462391 | SKN_EX |
| FAM47E | PD | 482730 | rs7699714 | -0.166873988 | 0.036323403 | 4.35E-06 | 0.84630625 | 0.78814921 | 0.90875466 | SKN_NEX |
| FAM47E | PD | 482730 | rs7699714 | -0.137361091 | 0.029899341 | 4.35E-06 | 0.871655422 | 0.82204199 | 0.92426322 | LIVER |
| FAM47E | PD | 482730 | rs7699714 | -0.166873988 | 0.036323403 | 4.35E-06 | 0.84630625 | 0.78814921 | 0.90875466 | SKN_NEX |
| FDFT1 | PD | 482730 | rs2686197 | 0.577978933 | 0.127478388 | 5.79E-06 | 1.782432373 | 1.38835736 | 2.28836268 | LUNG |
| GAK | PD | 482730 | rs56785826 | 0.959546903 | 0.171594995 | 2.25E-08 | 2.61051339 | 1.86492477 | 3.65418503 | SKN_EX |
| GAK | PD | 482730 | rs56785826 | 0.565924638 | 0.101203845 | 2.25E-08 | 1.761075388 | 1.44421382 | 2.14745662 | SKN_NEX |
| GAK | PD | 482730 | rs17165130 | 0.509005722 | 0.085473099 | 2.60E-09 | 1.663636255 | 1.40702627 | 1.96704614 | LUNG |
| GAK | PD | 482730 | rs56785826 | 0.565924638 | 0.101203845 | 2.25E-08 | 1.761075388 | 1.44421382 | 2.14745662 | SKN_NEX |
| GPNMB | PD | 482730 | rs858271 | 0.154099365 | 0.027092673 | 1.29E-08 | 1.166606801 | 1.10627413 | 1.23022983 | AD_COR |
| GPNMB | PD | 482730 | rs199348 | 0.428165963 | 0.07504834 | 1.16E-08 | 1.53444072 | 1.32454793 | 1.7775939 | AR_AO |

| exposure | outcome | samplesize | SNP | beta | standard error | p-value | odds ratio (OR) | OR_lci95 | OR_uci95 | tissue |
|---|---|---|---|---|---|---|---|---|---|---|
| GPNMB | PD | 482730 | rs858272 | 0.384116321 | 0.068156024 | 1.74E-08 | 1.468316228 | 1.28470679 | 1.678167 | SKN_EX |
| GPNMB | PD | 482730 | rs858271 | 0.428860928 | 0.07539933 | 1.29E-08 | 1.535507474 | 1.32455724 | 1.78005385 | SKN_NEX |
| GPNMB | PD | 482730 | rs10085838 | 0.523591724 | 0.100322203 | 1.80E-07 | 1.688079892 | 1.38674629 | 2.0548919 | BLOOD |
| GPNMB | PD | 482730 | rs1728313 | 0.350954329 | 0.061891029 | 1.42E-08 | 1.420422453 | 1.25815695 | 1.60361546 | LIVER |
| GPNMB | PD | 482730 | rs858271 | 0.428860928 | 0.07539933 | 1.29E-08 | 1.535507474 | 1.32455724 | 1.78005385 | SKN_NEX |
| HSD3B7 | PD | 482730 | rs9938550 | -0.231279237 | 0.039371256 | 4.25E-09 | 0.793517856 | 0.73458696 | 0.85717638 | SKN_EX |
| HSD3B7 | PD | 482730 | rs13708 | -0.257378626 | 0.043814222 | 4.25E-09 | 0.773075452 | 0.70945763 | 0.84239795 | SKN_NEX |
| HSD3B7 | PD | 482730 | rs9926533 | 0.491282559 | 0.104728498 | 2.72E-06 | 1.634411104 | 1.33111203 | 2.00681806 | BLOOD |
| HSD3B7 | PD | 482730 | rs4889599 | -0.353552219 | 0.057460085 | 7.60E-10 | 0.702189324 | 0.62739812 | 0.78589628 | LIVER |
| HSD3B7 | PD | 482730 | rs13708 | -0.257378626 | 0.043814222 | 4.25E-09 | 0.773075452 | 0.70945763 | 0.84239795 | SKN_NEX |
| IDUA | PD | 482730 | rs6599388 | -0.562335468 | 0.060003393 | 7.14E-21 | 0.56987658 | 0.50664632 | 0.64099808 | AR_AO |
| IDUA | PD | 482730 | rs3822019 | -0.303664027 | 0.049489205 | 8.46E-10 | 0.73810881 | 0.66987586 | 0.81329191 | AR_CO |
| IDUA | PD | 482730 | rs11248061 | -0.336265911 | 0.061323042 | 4.17E-08 | 0.714433105 | 0.63352294 | 0.80567669 | LUNG |
| IGSF9B | PD | 482730 | rs502834 | -0.317016684 | 0.061830762 | 2.94E-07 | 0.728318604 | 0.64519352 | 0.82215331 | AR_AO |
| IGSF9B | PD | 482730 | rs11223635 | -0.383344633 | 0.077298097 | 7.07E-07 | 0.681577965 | 0.58575779 | 0.79307272 | AR_CO |
| IGSF9B | PD | 482730 | rs329642 | -0.396738414 | 0.077368489 | 2.93E-07 | 0.672509922 | 0.57788485 | 0.78262926 | BLOOD |
| IGSF9B | PD | 482730 | rs329637 | -0.277929875 | 0.053246809 | 1.79E-07 | 0.757349929 | 0.68229469 | 0.84066156 | LUNG |
| IRF2BP1 | PD | 482730 | rs199525 | 0.976291642 | 0.119819789 | 3.70E-16 | 2.654593782 | 2.09896603 | 3.35730453 | AR_CO |
| KANSL1 | PD | 482730 | rs11656151 | 0.164554076 | 0.034313871 | 1.62E-06 | 1.178867316 | 1.10218976 | 1.26087921 | AD_COR |
| KANSL1 | PD | 482730 | rs11653367 | 0.207904382 | 0.043714302 | 1.97E-06 | 1.23109545 | 1.13000759 | 1.34122639 | LIVER |
| KANSL1 | PD | 482730 | rs11656151 | 0.194532045 | 0.04056507 | 1.62E-06 | 1.214742409 | 1.12190093 | 1.31526686 | LUNG |
| KANSL1-AS1 | PD | 480593 | rs2532387 | -0.200212202 | 0.022129564 | 1.47E-19 | 0.818557035 | 0.78381195 | 0.85484231 | AR_AO |
| KANSL1-AS1 | PD | 482730 | rs9468 | -0.196688075 | 0.020904848 | 5.02E-21 | 0.821446823 | 0.78846948 | 0.85580343 | AR_CO |
| KANSL1-AS1 | PD | 480593 | rs2950015 | -0.189124214 | 0.021269755 | 6.02E-19 | 0.827683691 | 0.79388796 | 0.8629181 | SKN_EX |
| KANSL1-AS1 | PD | 482730 | rs62060793 | -0.181286343 | 0.019336191 | 6.89E-21 | 0.834196458 | 0.8031729 | 0.86641835 | SKN_NEX |

| exposure | outcome | samplesize | SNP | beta | standard error | p-value | odds ratio (OR) | OR_lci95 | OR_uci95 | tissue |
|---|---|---|---|---|---|---|---|---|---|---|
| KANSL1-AS1 | PD | 480593 | rs2732713 | -0.190121247 | 0.021946143 | 4.59E-18 | 0.826858874 | 0.7920461 | 0.86320178 | BLOOD |
| KANSL1-AS1 | PD | 482730 | rs9468 | -0.19383378 | 0.020601482 | 5.02E-21 | 0.823794824 | 0.79119352 | 0.85773947 | LIVER |
| KANSL1-AS1 | PD | 482730 | rs2696566 | -0.178681282 | 0.019783692 | 1.69E-19 | 0.836372424 | 0.80456194 | 0.86944061 | LUNG |
| KANSL1-AS1 | PD | 482730 | rs62060793 | -0.181286343 | 0.019336191 | 6.89E-21 | 0.834196458 | 0.8031729 | 0.86641835 | SKN_NEX |
| KAT8 | PD | 482730 | rs7187995 | -0.197313783 | 0.038595443 | 3.18E-07 | 0.820932998 | 0.76112258 | 0.88544342 | AD_COR |
| KAT8 | PD | 482730 | rs2855475 | -0.201498445 | 0.039631738 | 3.69E-07 | 0.817504849 | 0.75640627 | 0.88353865 | AR_AO |
| KAT8 | PD | 482730 | rs9972727 | -0.254877927 | 0.049759248 | 3.02E-07 | 0.7750111 | 0.70299461 | 0.85440514 | AR_CO |
| KAT8 | PD | 482730 | rs4527034 | -0.206072188 | 0.039526777 | 1.85E-07 | 0.813774329 | 0.75310948 | 0.87932589 | SKN_EX |
| KAT8 | PD | 482730 | rs4527034 | -0.229205271 | 0.043963942 | 1.85E-07 | 0.795165292 | 0.72951555 | 0.86672291 | SKN_NEX |
| KAT8 | PD | 482730 | rs4527034 | -0.293616957 | 0.05631877 | 1.85E-07 | 0.745562019 | 0.66764296 | 0.83257484 | LIVER |
| KAT8 | PD | 482730 | rs7187995 | -0.291984893 | 0.057113528 | 3.18E-07 | 0.746779818 | 0.66769259 | 0.83523482 | LUNG |
| KAT8 | PD | 482730 | rs4527034 | -0.229205271 | 0.043963942 | 1.85E-07 | 0.795165292 | 0.72951555 | 0.86672291 | SKN_NEX |
| KLHL7-AS1 | PD | 482730 | rs10231555 | -0.082677225 | 0.016553941 | 5.90E-07 | 0.920648261 | 0.89125656 | 0.95100924 | AD_COR |
| KLHL7-AS1 | PD | 482730 | rs2014768 | -0.086076634 | 0.017215327 | 5.73E-07 | 0.917523915 | 0.88708127 | 0.94901128 | AR_AO |
| KLHL7-AS1 | PD | 482730 | rs7776649 | -0.092697096 | 0.018278301 | 3.95E-07 | 0.911469546 | 0.87939372 | 0.94471534 | AR_CO |
| KLHL7-AS1 | PD | 482730 | rs10256524 | -0.084848606 | 0.016799835 | 4.41E-07 | 0.918651352 | 0.88889489 | 0.94940393 | SKN_EX |
| KLHL7-AS1 | PD | 482730 | rs10235786 | -0.080684514 | 0.016082993 | 5.26E-07 | 0.922484677 | 0.89385905 | 0.95202703 | SKN_NEX |
| KLHL7-AS1 | PD | 482730 | rs10488077 | -0.107553989 | 0.021342932 | 4.67E-07 | 0.898028037 | 0.8612365 | 0.93639129 | LIVER |
| KLHL7-AS1 | PD | 482730 | rs1468592 | -0.095588643 | 0.019160545 | 6.07E-07 | 0.908837796 | 0.87533964 | 0.94361789 | LUNG |
| KLHL7-AS1 | PD | 482730 | rs10235786 | -0.080684514 | 0.016082993 | 5.26E-07 | 0.922484677 | 0.89385905 | 0.95202703 | SKN_NEX |
| LINC02210 | PD | 480593 | rs77692262 | -0.406394653 | 0.043873073 | 1.99E-20 | 0.666047258 | 0.61116649 | 0.72585615 | AD_COR |
| LINC02210 | PD | 480593 | rs2532387 | -0.183335577 | 0.020264182 | 1.47E-19 | 0.832488745 | 0.80007215 | 0.86621877 | AR_AO |
| LINC02210 | PD | 480593 | rs62054846 | -0.195542569 | 0.021135506 | 2.21E-20 | 0.822388335 | 0.78901641 | 0.85717175 | AR_CO |
| LINC02210 | PD | 480593 | rs56328224 | -0.200881152 | 0.02279624 | 1.23E-18 | 0.818009645 | 0.78226495 | 0.85538765 | SKN_EX |
| LINC02210 | PD | 480593 | rs4383188 | -0.212023756 | 0.022594746 | 6.37E-21 | 0.80894548 | 0.77390244 | 0.8455753 | SKN_NEX |

| exposure | outcome | samplesize | SNP | beta | standard error | p-value | odds ratio (OR) | OR_lci95 | OR_uci95 | tissue |
|---|---|---|---|---|---|---|---|---|---|---|
| LINC02210 | PD | 482730 | rs199530 | 0.602039206 | 0.070755123 | 1.76E-17 | 1.825838267 | 1.5894041 | 2.09744355 | LIVER |
| LINC02210 | PD | 480593 | rs78929339 | -0.209766457 | 0.022223108 | 3.76E-21 | 0.810773574 | 0.77621654 | 0.84686908 | LUNG |
| LINC02210 | PD | 480593 | rs4383188 | -0.212023756 | 0.022594746 | 6.37E-21 | 0.80894548 | 0.77390244 | 0.8455753 | SKN_NEX |
| LRRC37A | PD | 480593 | rs111676341 | -0.541674627 | 0.05762496 | 5.46E-21 | 0.581773183 | 0.51963972 | 0.65133596 | BLOOD |
| LRRC37A4P | PD | 480593 | rs56328224 | 0.188392029 | 0.021378959 | 1.23E-18 | 1.207306722 | 1.15776251 | 1.25897109 | AR_AO |
| LRRC37A4P | PD | 480593 | rs56328224 | 0.19185852 | 0.021772341 | 1.23E-18 | 1.211499102 | 1.16088742 | 1.26431732 | AR_CO |
| LRRC37A4P | PD | 480593 | rs241039 | 0.210801349 | 0.022742397 | 1.88E-20 | 1.234667062 | 1.18084024 | 1.2909475 | SKN_EX |
| LRRC37A4P | PD | 480593 | rs241039 | 0.212900038 | 0.022968815 | 1.88E-20 | 1.237260967 | 1.18279604 | 1.29423387 | SKN_NEX |
| LRRC37A4P | PD | 480593 | rs650927 | 0.214862498 | 0.023020982 | 1.03E-20 | 1.239691425 | 1.18499834 | 1.29690885 | BLOOD |
| LRRC37A4P | PD | 480593 | rs34739559 | 0.223576978 | 0.024342853 | 4.13E-20 | 1.250541901 | 1.19227707 | 1.31165404 | LIVER |
| LRRC37A4P | PD | 480593 | rs241039 | 0.212900038 | 0.022968815 | 1.88E-20 | 1.237260967 | 1.18279604 | 1.29423387 | SKN_NEX |
| LRRK2 | PD | 482730 | rs1491942 | 0.32222143 | 0.055758783 | 7.52E-09 | 1.380190358 | 1.23730325 | 1.53957845 | AR_CO |
| MALSU1 | PD | 482730 | rs466240 | 0.526557198 | 0.097286664 | 6.22E-08 | 1.693093278 | 1.39916459 | 2.04876887 | LIVER |
| MAPK8IP1P1 | PD | 482730 | rs9468 | -0.205860826 | 0.021879767 | 5.02E-21 | 0.813946349 | 0.77977866 | 0.84961117 | SKN_EX |
| MAPK8IP1P1 | PD | 482730 | rs9468 | -0.200866547 | 0.021348954 | 5.02E-21 | 0.818021591 | 0.7844986 | 0.85297708 | SKN_NEX |
| MAPK8IP1P1 | PD | 482730 | rs8712 | -0.21478338 | 0.022751329 | 3.71E-21 | 0.806716172 | 0.77153288 | 0.84350388 | BLOOD |
| MAPK8IP1P1 | PD | 480593 | rs17571739 | -0.229940856 | 0.025106355 | 5.25E-20 | 0.794580595 | 0.75642695 | 0.83465868 | LIVER |
| MAPK8IP1P1 | PD | 482730 | rs2668645 | -0.20620241 | 0.022695511 | 1.03E-19 | 0.813668365 | 0.77826701 | 0.85068004 | LUNG |
| MAPK8IP1P1 | PD | 482730 | rs9468 | -0.200866547 | 0.021348954 | 5.02E-21 | 0.818021591 | 0.7844986 | 0.85297708 | SKN_NEX |
| MAPK8IP1P2 | PD | 480593 | rs448830 | -0.218085051 | 0.023338472 | 9.24E-21 | 0.804057053 | 0.76810529 | 0.84169156 | AR_AO |
| MAPK8IP1P2 | PD | 480593 | rs62056841 | -0.206874039 | 0.022179799 | 1.09E-20 | 0.813122065 | 0.77853102 | 0.84925003 | AR_CO |
| MAPK8IP1P2 | PD | 480593 | rs55974014 | -0.207865095 | 0.022233723 | 8.84E-21 | 0.812316615 | 0.77767764 | 0.84849847 | SKN_EX |
| MAPK8IP1P2 | PD | 480593 | rs74398257 | -0.20741672 | 0.02207958 | 5.77E-21 | 0.812680919 | 0.7782615 | 0.84862258 | SKN_NEX |
| MAPK8IP1P2 | PD | 480593 | rs681485 | -0.207988193 | 0.022351951 | 1.34E-20 | 0.812216627 | 0.77740174 | 0.84859065 | BLOOD |
| MAPK8IP1P2 | PD | 480593 | rs111511018 | -0.173388457 | 0.020192879 | 8.96E-18 | 0.840810932 | 0.80818321 | 0.87475589 | LIVER |

| exposure | outcome | samplesize | SNP | beta | standard error | p-value | odds ratio (OR) | OR_lci95 | OR_uci95 | tissue |
|---|---|---|---|---|---|---|---|---|---|---|
| MAPK8IP1P2 | PD | 480593 | rs594591 | -0.216547529 | 0.023201521 | 1.03E-20 | 0.805294259 | 0.7694937 | 0.84276043 | LUNG |
| MAPK8IP1P2 | PD | 480593 | rs74398257 | -0.20741672 | 0.02207958 | 5.77E-21 | 0.812680919 | 0.7782615 | 0.84862258 | SKN_NEX |
| MAPT | PD | 482730 | rs3785884 | -0.473173139 | 0.049912778 | 2.54E-21 | 0.623022192 | 0.56495896 | 0.68705283 | SKN_EX |
| MAPT | PD | 482730 | rs2471737 | -0.523567869 | 0.069437724 | 4.70E-14 | 0.592403156 | 0.51702409 | 0.67877205 | SKN_NEX |
| MAPT | PD | 480593 | rs7210219 | -0.440149688 | 0.046429292 | 2.54E-21 | 0.643940024 | 0.58792782 | 0.70528855 | LUNG |
| MAPT | PD | 482730 | rs2471737 | -0.523567869 | 0.069437724 | 4.70E-14 | 0.592403156 | 0.51702409 | 0.67877205 | SKN_NEX |
| MAPT-IT1 | PD | 480593 | rs7210219 | 0.600177636 | 0.063309877 | 2.54E-21 | 1.822442503 | 1.60976833 | 2.06321407 | LIVER |
| MCCC1 | PD | 482730 | rs10937106 | 0.566376924 | 0.09703482 | 5.32E-09 | 1.761872077 | 1.45672196 | 2.13094421 | BLOOD |
| MMRN1 | PD | 482730 | rs3775467 | 0.359802472 | 0.057146084 | 3.05E-10 | 1.43304632 | 1.28119873 | 1.60289088 | AD_COR |
| MMRN1 | PD | 480593 | rs74739978 | 1.096606122 | 0.165142798 | 3.13E-11 | 2.993987534 | 2.1660955 | 4.13830385 | SKN_EX |
| MMRN1 | PD | 482730 | rs61611959 | 0.673684302 | 0.102689962 | 5.37E-11 | 1.9614506 | 1.603858 | 2.39877125 | LIVER |
| NCOR1 | PD | 482730 | rs9898313 | 0.774180467 | 0.148781051 | 1.96E-07 | 2.168813985 | 1.62023242 | 2.9031354 | SKN_NEX |
| NCOR1 | PD | 482730 | rs9898313 | 0.774180467 | 0.148781051 | 1.96E-07 | 2.168813985 | 1.62023242 | 2.9031354 | SKN_NEX |
| NSF | PD | 480593 | rs12938031 | 0.742446374 | 0.126930265 | 4.94E-09 | 2.101069233 | 1.63830645 | 2.6945459 | AR_AO |
| NUCKS1 | PD | 482730 | rs823116 | -0.877221027 | 0.148962061 | 3.89E-09 | 0.415937186 | 0.31061948 | 0.5569636 | LUNG |
| NUPL2 | PD | 482730 | rs1637221 | 0.14462791 | 0.024761199 | 5.19E-09 | 1.1556095 | 1.1008647 | 1.2130767 | AD_COR |
| NUPL2 | PD | 482730 | rs870476 | 0.247957271 | 0.04937323 | 5.11E-07 | 1.281405178 | 1.16321269 | 1.41160704 | AR_AO |
| NUPL2 | PD | 482730 | rs4365988 | 0.198630609 | 0.040080424 | 7.20E-07 | 1.219731325 | 1.12757914 | 1.31941471 | AR_CO |
| NUPL2 | PD | 482730 | rs1006709 | 0.665775508 | 0.116871835 | 1.22E-08 | 1.945999074 | 1.54760212 | 2.44695478 | SKN_EX |
| NUPL2 | PD | 482730 | rs55788996 | 0.546710933 | 0.110935739 | 8.30E-07 | 1.727561598 | 1.38996266 | 2.14715773 | BLOOD |
| NUPL2 | PD | 482730 | rs6955115 | 0.259032954 | 0.05256162 | 8.30E-07 | 1.295676502 | 1.16884044 | 1.43627611 | LUNG |
| PLEKHM1 | PD | 482730 | rs8070723 | 3.409047829 | 0.364688838 | 8.95E-21 | 30.23644029 | 14.7945328 | 61.7959576 | BLOOD |
| PLEKHM1 | PD | 480593 | rs56168933 | -0.787586783 | 0.097342187 | 5.92E-16 | 0.454941344 | 0.37592055 | 0.5505728 | LUNG |
| PRSS36 | PD | 482730 | rs113448632 | -0.210604081 | 0.038575031 | 4.77E-08 | 0.810094735 | 0.75110401 | 0.87371851 | AD_COR |
| PRSS53 | PD | 482730 | rs11647284 | 0.592762134 | 0.121489536 | 1.07E-06 | 1.808978161 | 1.42567109 | 2.29534148 | AR_AO |

| exposure | outcome | samplesize | SNP | beta | standard error | p-value | odds ratio (OR) | OR_lci95 | OR_uci95 | tissue |
|---|---|---|---|---|---|---|---|---|---|---|
| PRSS53 | PD | 482730 | rs59061704 | -0.37359239 | 0.075214397 | 6.80E-07 | 0.688257395 | 0.59391884 | 0.79758077 | SKN_EX |
| PRSS53 | PD | 482730 | rs56813533 | -0.126003066 | 0.025873019 | 1.12E-06 | 0.881612144 | 0.83801927 | 0.92747267 | LIVER |
| RAB29 | PD | 482730 | rs10900524 | 0.417326955 | 0.090280699 | 3.79E-06 | 1.517898716 | 1.27172823 | 1.81172082 | AD_COR |
| RAB29 | PD | 482730 | rs823118 | 0.196548576 | 0.03364345 | 5.15E-09 | 1.217194445 | 1.13952033 | 1.30016312 | AR_AO |
| RAB29 | PD | 482730 | rs708723 | 0.202453949 | 0.035362232 | 1.03E-08 | 1.224403698 | 1.14241446 | 1.31227717 | AR_CO |
| RAB29 | PD | 482730 | rs823144 | 0.233681788 | 0.045287168 | 2.47E-07 | 1.263242449 | 1.15594586 | 1.38049846 | SKN_EX |
| RAB29 | PD | 482730 | rs863725 | 0.237861779 | 0.047054754 | 4.30E-07 | 1.268533843 | 1.15677327 | 1.39109206 | SKN_NEX |
| RAB29 | PD | 482730 | rs708723 | 0.312894084 | 0.054652593 | 1.03E-08 | 1.367376696 | 1.22847677 | 1.52198159 | BLOOD |
| RAB29 | PD | 482730 | rs863725 | 0.280696455 | 0.055528478 | 4.30E-07 | 1.324051633 | 1.18751233 | 1.47629013 | LIVER |
| RAB29 | PD | 482730 | rs1775158 | 0.213805219 | 0.042379627 | 4.54E-07 | 1.238381418 | 1.13967274 | 1.3456394 | LUNG |
| RAB29 | PD | 482730 | rs863725 | 0.237861779 | 0.047054754 | 4.30E-07 | 1.268533843 | 1.15677327 | 1.39109206 | SKN_NEX |
| RNF40 | PD | 482730 | rs8046391 | -0.432326015 | 0.084632943 | 3.25E-07 | 0.648997759 | 0.54979677 | 0.7660978 | AD_COR |
| RNF40 | PD | 482730 | rs7204278 | -0.52729001 | 0.111747297 | 2.37E-06 | 0.590202247 | 0.47411057 | 0.73472037 | BLOOD |
| RNF40 | PD | 482730 | rs8058961 | -0.581825209 | 0.118060266 | 8.30E-07 | 0.558877367 | 0.44342645 | 0.70438719 | LIVER |
| RNF40 | PD | 482730 | rs3747486 | -0.632931337 | 0.133708425 | 2.20E-06 | 0.531032881 | 0.40860766 | 0.69013861 | LUNG |
| RP11-1072A3.3 | PD | 482730 | rs57576577 | -0.445972453 | 0.090964613 | 9.45E-07 | 0.640201407 | 0.53565601 | 0.7651512 | SKN_EX |
| RP11-1072A3.3 | PD | 482730 | rs9929899 | 0.492354933 | 0.101098403 | 1.12E-06 | 1.636164745 | 1.34205506 | 1.9947282 | BLOOD |
| RP11-115D19.1 | PD | 27693 | rs356229 | -0.663739317 | 0.117610904 | 1.67E-08 | 0.514922273 | 0.40891143 | 0.64841656 | SKN_EX |
| RP11-115D19.1 | PD | 27693 | rs356229 | -0.607605759 | 0.107664351 | 1.67E-08 | 0.544653341 | 0.44103639 | 0.67261402 | SKN_NEX |
| RP11-115D19.1 | PD | 27693 | rs356229 | -0.607605759 | 0.107664351 | 1.67E-08 | 0.544653341 | 0.44103639 | 0.67261402 | SKN_NEX |
| RP11-115L11.1 | PD | 482730 | rs4698119 | -0.363775753 | 0.061645425 | 3.61E-09 | 0.69504704 | 0.61594304 | 0.78431016 | AR_AO |
| RP11-115L11.1 | PD | 482730 | rs4389574 | -0.474509956 | 0.064256557 | 1.53E-13 | 0.622189882 | 0.54856319 | 0.70569856 | SKN_EX |
| RP11-196G11.2 | PD | 482730 | rs11640957 | -0.274789896 | 0.055568623 | 7.61E-07 | 0.759731729 | 0.6813328 | 0.84715179 | AD_COR |
| RP11-196G11.2 | PD | 482730 | rs56284083 | -0.21296043 | 0.04145326 | 2.79E-07 | 0.808188116 | 0.74512088 | 0.87659338 | AR_AO |
| RP11-196G11.2 | PD | 482730 | rs7196726 | -0.242399708 | 0.048912316 | 7.20E-07 | 0.784742447 | 0.71300429 | 0.86369847 | AR_CO |

| exposure | outcome | samplesize | SNP | beta | standard error | p-value | odds ratio (OR) | OR_lci95 | OR_uci95 | tissue |
|---|---|---|---|---|---|---|---|---|---|---|
| RP11-196G11.2 | PD | 480593 | rs55979739 | -0.191153175 | 0.037028658 | 2.44E-07 | 0.826006055 | 0.76818142 | 0.88818342 | SKN_EX |
| RP11-196G11.2 | PD | 482730 | rs732172 | -0.152430855 | 0.029675368 | 2.80E-07 | 0.858618261 | 0.81010243 | 0.91003963 | SKN_NEX |
| RP11-196G11.2 | PD | 482730 | rs8047803 | -0.268934107 | 0.05457071 | 8.30E-07 | 0.764193609 | 0.68667601 | 0.85046203 | BLOOD |
| RP11-196G11.2 | PD | 482730 | rs6565217 | -0.319239513 | 0.064997021 | 9.03E-07 | 0.726701474 | 0.63977823 | 0.82543451 | LIVER |
| RP11-196G11.2 | PD | 482730 | rs12445650 | -0.182176749 | 0.035537158 | 2.95E-07 | 0.833454016 | 0.77737721 | 0.89357597 | LUNG |
| RP11-196G11.2 | PD | 482730 | rs732172 | -0.152430855 | 0.029675368 | 2.80E-07 | 0.858618261 | 0.81010243 | 0.91003963 | SKN_NEX |
| RP11-196G11.3 | PD | 482730 | rs2884737 | -0.316592926 | 0.069708534 | 5.58E-06 | 0.7286273 | 0.63557724 | 0.83530012 | SKN_NEX |
| RP11-196G11.3 | PD | 482730 | rs56813533 | -0.117030774 | 0.024030681 | 1.12E-06 | 0.889557817 | 0.84863092 | 0.93245849 | LIVER |
| RP11-196G11.3 | PD | 482730 | rs2884737 | -0.316592926 | 0.069708534 | 5.58E-06 | 0.7286273 | 0.63557724 | 0.83530012 | SKN_NEX |
| RP11-196G11.4 | PD | 482730 | rs7187995 | -0.738904996 | 0.144533065 | 3.18E-07 | 0.477636643 | 0.35980617 | 0.63405461 | BLOOD |
| RP11-196G11.6 | PD | 482730 | rs12597511 | 0.183751004 | 0.034869594 | 1.37E-07 | 1.201716564 | 1.12232969 | 1.28671879 | LIVER |
| RP11-231G3.1 | PD | 482730 | rs4647102 | 0.412807769 | 0.08153168 | 4.12E-07 | 1.511054526 | 1.28789059 | 1.77288801 | BLOOD |
| RP11-242D8.1 | PD | 480593 | rs62054807 | -1.020121698 | 0.121378838 | 4.30E-17 | 0.360551059 | 0.28421502 | 0.45738986 | LIVER |
| RP11-259G18.1 | PD | 482730 | rs1991556 | -0.324982526 | 0.035887641 | 1.36E-19 | 0.722539979 | 0.67346295 | 0.77519339 | AR_AO |
| RP11-259G18.1 | PD | 480593 | rs10451283 | -0.509014641 | 0.054249183 | 6.42E-21 | 0.601087574 | 0.54045547 | 0.66852182 | AR_CO |
| RP11-259G18.1 | PD | 482730 | rs17572147 | -0.297411818 | 0.031870051 | 1.04E-20 | 0.742738076 | 0.69776206 | 0.79061313 | SKN_EX |
| RP11-259G18.1 | PD | 482730 | rs1991556 | -0.305870804 | 0.033777144 | 1.36E-19 | 0.736481764 | 0.68930323 | 0.78688938 | SKN_NEX |
| RP11-259G18.1 | PD | 482730 | rs55881134 | -0.667378987 | 0.071063503 | 5.93E-21 | 0.513051532 | 0.44634483 | 0.58972761 | LUNG |
| RP11-259G18.1 | PD | 482730 | rs1991556 | -0.305870804 | 0.033777144 | 1.36E-19 | 0.736481764 | 0.68930323 | 0.78688938 | SKN_NEX |
| RP11-259G18.3 | PD | 480593 | rs62071573 | -0.19530329 | 0.022063218 | 8.60E-19 | 0.822585138 | 0.78777151 | 0.85893727 | AR_AO |
| RP11-259G18.3 | PD | 480593 | rs2532387 | -0.199519757 | 0.022053028 | 1.47E-19 | 0.819124037 | 0.78447255 | 0.85530613 | AR_CO |
| RP11-259G18.3 | PD | 480593 | rs73984689 | -0.199188068 | 0.020975343 | 2.17E-21 | 0.819395777 | 0.78639211 | 0.85378455 | SKN_EX |
| RP11-259G18.3 | PD | 482730 | rs113417378 | -0.18106584 | 0.02001459 | 1.47E-19 | 0.834380421 | 0.80228254 | 0.86776248 | SKN_NEX |
| RP11-259G18.3 | PD | 480593 | rs2696673 | -0.190335581 | 0.021510794 | 8.88E-19 | 0.826681669 | 0.79255234 | 0.8622807 | BLOOD |
| RP11-259G18.3 | PD | 482730 | rs9468 | -0.189021977 | 0.020090062 | 5.02E-21 | 0.827768315 | 0.79580707 | 0.86101319 | LIVER |

| exposure | outcome | samplesize | SNP | beta | standard error | p-value | odds ratio (OR) | OR_lci95 | OR_uci95 | tissue |
|---|---|---|---|---|---|---|---|---|---|---|
| RP11-259G18.3 | PD | 480593 | rs62071573 | -0.191167291 | 0.021595979 | 8.60E-19 | 0.825994395 | 0.79176123 | 0.86170769 | LUNG |
| RP11-259G18.3 | PD | 482730 | rs113417378 | -0.18106584 | 0.02001459 | 1.47E-19 | 0.834380421 | 0.80228254 | 0.86776248 | SKN_NEX |
| RP11-2C24.7 | PD | 482730 | rs7200879 | -0.496160577 | 0.082826806 | 2.09E-09 | 0.608863863 | 0.51762661 | 0.71618266 | AR_AO |
| RP11-669E14.4 | PD | 480593 | rs111985258 | 0.395906259 | 0.042701998 | 1.84E-20 | 1.485730038 | 1.36644208 | 1.61543162 | LIVER |
| RP11-707O23.1 | PD | 13839 | rs440778 | -0.210909835 | 0.033450263 | 2.88E-10 | 0.809847083 | 0.75845459 | 0.86472191 | AR_AO |
| RP11-707O23.1 | PD | 480593 | rs241029 | -0.231856537 | 0.024802403 | 8.92E-21 | 0.79305989 | 0.75542918 | 0.83256513 | SKN_EX |
| RP11-707O23.1 | PD | 480593 | rs241029 | -0.222588735 | 0.023810998 | 8.92E-21 | 0.800443976 | 0.76394591 | 0.83868576 | SKN_NEX |
| RP11-707O23.1 | PD | 480593 | rs62055697 | -0.203201693 | 0.021630889 | 5.77E-21 | 0.81611362 | 0.78223644 | 0.85145796 | LIVER |
| RP11-707O23.1 | PD | 480593 | rs241029 | -0.222588735 | 0.023810998 | 8.92E-21 | 0.800443976 | 0.76394591 | 0.83868576 | SKN_NEX |
| RP11-927P21.1 | PD | 482730 | rs72836333 | 0.154197293 | 0.035020474 | 1.07E-05 | 1.16672105 | 1.08932383 | 1.2496174 | LIVER |
| SLC45A3 | PD | 482730 | rs4951254 | -0.439419471 | 0.071627554 | 8.53E-10 | 0.644410412 | 0.56000505 | 0.74153756 | AR_AO |
| SMIM15 | PD | 481731 | rs34613 | -0.894723352 | 0.194129019 | 4.05E-06 | 0.408720655 | 0.27937055 | 0.59796056 | SKN_EX |
| SNCA | PD | 482730 | rs356224 | 0.8722243 | 0.092382067 | 3.68E-21 | 2.392225968 | 1.9960205 | 2.86707731 | SKN_NEX |
| SNCA | PD | 482730 | rs2583990 | -1.730825741 | 0.182618308 | 2.60E-21 | 0.177138079 | 0.1238409 | 0.25337267 | BLOOD |
| SNCA | PD | 482730 | rs356224 | 0.8722243 | 0.092382067 | 3.68E-21 | 2.392225968 | 1.9960205 | 2.86707731 | SKN_NEX |
| STBD1 | PD | 482730 | rs4859442 | -0.377131383 | 0.074376259 | 3.97E-07 | 0.685825962 | 0.59279369 | 0.7934586 | LUNG |
| STX1B | PD | 482730 | rs7500176 | -0.185390036 | 0.037279518 | 6.59E-07 | 0.830780187 | 0.77224155 | 0.89375626 | AR_AO |
| STX1B | PD | 482730 | rs3751855 | -0.294492056 | 0.060093644 | 9.56E-07 | 0.744909864 | 0.66214177 | 0.83802402 | AR_CO |
| STX1B | PD | 482730 | rs58726213 | -0.516557699 | 0.100981204 | 3.13E-07 | 0.596570593 | 0.48944614 | 0.72714124 | SKN_EX |
| STX1B | PD | 482730 | rs9673641 | -0.58758898 | 0.11790226 | 6.24E-07 | 0.555665391 | 0.44101455 | 0.70012209 | SKN_NEX |
| STX1B | PD | 482730 | rs4889606 | 0.48117831 | 0.09970866 | 1.39E-06 | 1.617979761 | 1.33075884 | 1.96719227 | BLOOD |
| STX1B | PD | 482730 | rs9673641 | -0.58758898 | 0.11790226 | 6.24E-07 | 0.555665391 | 0.44101455 | 0.70012209 | SKN_NEX |
| STX4 | PD | 482730 | rs11640957 | 0.434087649 | 0.087782169 | 7.61E-07 | 1.543554153 | 1.2995715 | 1.83334231 | AD_COR |
| STX4 | PD | 482730 | rs12445568 | -1.056001597 | 0.197192135 | 8.55E-08 | 0.347843854 | 0.23633661 | 0.51196193 | SKN_EX |
| TMEM163 | PD | 482730 | rs6753334 | 0.254626875 | 0.037470176 | 1.08E-11 | 1.289980207 | 1.1986373 | 1.38828395 | AR_AO |

| exposure | outcome | samplesize | SNP | beta | standard error | p-value | odds ratio (OR) | OR_lci95 | OR_uci95 | tissue |
|---|---|---|---|---|---|---|---|---|---|---|
| TMEM163 | PD | 27693 | rs6758044 | 0.277843683 | 0.049257606 | 1.69E-08 | 1.320279798 | 1.19877329 | 1.45410209 | AR_CO |
| TMEM163 | PD | 482730 | rs1942041 | 0.720685136 | 0.106835234 | 1.52E-11 | 2.055841259 | 1.6674376 | 2.53471751 | LUNG |
| TMEM175 | PD | 480593 | rs2306244 | -0.282977981 | 0.057666192 | 9.24E-07 | 0.753536379 | 0.67300419 | 0.84370511 | AD_COR |
| TMEM175 | PD | 482730 | rs56039006 | -0.5355562 | 0.085253336 | 3.34E-10 | 0.585343632 | 0.49526974 | 0.69179912 | AR_AO |
| TMEM175 | PD | 482730 | rs4690340 | -0.390287997 | 0.070560542 | 3.18E-08 | 0.676861912 | 0.58943746 | 0.77725303 | AR_CO |
| TMEM175 | PD | 482730 | rs2290402 | -0.618315603 | 0.101049418 | 9.42E-10 | 0.538851313 | 0.44203224 | 0.65687684 | SKN_EX |
| TMEM175 | PD | 482730 | rs56039006 | -0.627895872 | 0.099952569 | 3.34E-10 | 0.533713622 | 0.43875991 | 0.64921662 | SKN_NEX |
| TMEM175 | PD | 482730 | rs3822019 | -0.530857728 | 0.08651577 | 8.46E-10 | 0.588100323 | 0.49637249 | 0.69677912 | LUNG |
| TMEM175 | PD | 482730 | rs56039006 | -0.627895872 | 0.099952569 | 3.34E-10 | 0.533713622 | 0.43875991 | 0.64921662 | SKN_NEX |
| TTC19 | PD | 482730 | rs2285583 | -0.490372429 | 0.09584552 | 3.12E-07 | 0.612398277 | 0.50751468 | 0.73895725 | AD_COR |
| TTC19 | PD | 482730 | rs3760298 | -0.367503763 | 0.070966244 | 2.24E-07 | 0.692460721 | 0.60254218 | 0.79579798 | AR_AO |
| TTC19 | PD | 482730 | rs178811 | -0.233639974 | 0.04640794 | 4.79E-07 | 0.791646778 | 0.72281676 | 0.86703112 | SKN_EX |
| TTC19 | PD | 482730 | rs11078321 | -0.244637564 | 0.047458558 | 2.54E-07 | 0.78298827 | 0.71344043 | 0.8593158 | SKN_NEX |
| TTC19 | PD | 482730 | rs758853 | -0.316625986 | 0.061852518 | 3.07E-07 | 0.728603212 | 0.64541812 | 0.82250966 | BLOOD |
| TTC19 | PD | 482730 | rs2779211 | -0.218871582 | 0.044031812 | 6.67E-07 | 0.803424886 | 0.73699517 | 0.8758423 | LIVER |
| TTC19 | PD | 482730 | rs758853 | -0.234302164 | 0.045770655 | 3.07E-07 | 0.791122731 | 0.72324109 | 0.86537557 | LUNG |
| TTC19 | PD | 482730 | rs11078321 | -0.244637564 | 0.047458558 | 2.54E-07 | 0.78298827 | 0.71344043 | 0.8593158 | SKN_NEX |
| VKORC1 | PD | 482730 | rs8047803 | -0.440697288 | 0.089424001 | 8.30E-07 | 0.643587499 | 0.54011763 | 0.766879 | SKN_EX |
| VKORC1 | PD | 482730 | rs4468641 | -0.48179439 | 0.097872935 | 8.54E-07 | 0.617674048 | 0.50985682 | 0.74829093 | SKN_NEX |
| VKORC1 | PD | 482730 | rs7187995 | 0.773752874 | 0.151349463 | 3.18E-07 | 2.167886813 | 1.61140735 | 2.91653953 | BLOOD |
| VKORC1 | PD | 482730 | rs9796794 | -0.089190547 | 0.018187473 | 9.39E-07 | 0.914671269 | 0.88263988 | 0.94786509 | LIVER |
| VKORC1 | PD | 482730 | rs4468641 | -0.48179439 | 0.097872935 | 8.54E-07 | 0.617674048 | 0.50985682 | 0.74829093 | SKN_NEX |
| WNT3 | PD | 482730 | rs199523 | -0.312226395 | 0.038878438 | 9.68E-16 | 0.73181583 | 0.67812195 | 0.7897612 | AR_AO |
| WNT3 | PD | 480593 | rs199524 | -0.38304767 | 0.046424134 | 1.57E-16 | 0.681780398 | 0.62248299 | 0.74672644 | AR_CO |
| WNT3 | PD | 27693 | rs199498 | -0.561097871 | 0.107333497 | 1.72E-07 | 0.570582294 | 0.46233223 | 0.70417793 | BLOOD |

| exposure | outcome | samplesize | SNP | beta | standard error | p-value | odds ratio (OR) | OR_lci95 | OR_uci95 | tissue |
|---|---|---|---|---|---|---|---|---|---|---|
| WNT3 | PD | 482730 | rs199523 | -0.483526737 | 0.06020876 | 9.68E-16 | 0.616604949 | 0.54796936 | 0.69383745 | LUNG |
| ZNF646 | PD | 482730 | rs57434408 | -0.378103021 | 0.076716555 | 8.28E-07 | 0.685159911 | 0.58950772 | 0.79633242 | SKN_EX |
| ZNF646 | PD | 482730 | rs72800847 | -0.356786975 | 0.069543224 | 2.89E-07 | 0.699921583 | 0.61073527 | 0.80213187 | SKN_NEX |
| ZNF646 | PD | 482730 | rs4468641 | -0.28268197 | 0.057424732 | 8.54E-07 | 0.753759468 | 0.67352212 | 0.84355557 | LIVER |
| ZNF646 | PD | 482730 | rs72800847 | -0.356786975 | 0.069543224 | 2.89E-07 | 0.699921583 | 0.61073527 | 0.80213187 | SKN_NEX |
| ZNF668 | PD | 482730 | rs12716981 | 0.531477934 | 0.101396648 | 1.59E-07 | 1.701445075 | 1.39478531 | 2.07552755 | SKN_EX |
| ZNF668 | PD | 482730 | rs2288004 | 0.285388071 | 0.058262617 | 9.67E-07 | 1.330278171 | 1.18672017 | 1.49120244 | BLOOD |
| ZNF668 | PD | 482730 | rs34649473 | 0.494688313 | 0.100875382 | 9.39E-07 | 1.639986996 | 1.34577838 | 1.99851431 | LUNG |
| ZSWIM7 | PD | 482730 | rs1860643 | 0.1181101 | 0.022825772 | 2.29E-07 | 1.125368008 | 1.07613034 | 1.17685852 | AD_COR |
| ZSWIM7 | PD | 482730 | rs1045599 | 0.259552142 | 0.050737311 | 3.13E-07 | 1.296349376 | 1.17363646 | 1.4318929 | AR_AO |
| ZSWIM7 | PD | 482730 | rs4792708 | -0.201058016 | 0.040116653 | 5.39E-07 | 0.817864981 | 0.7560206 | 0.88476839 | SKN_EX |
| ZSWIM7 | PD | 482730 | rs4792712 | -0.229090774 | 0.04512394 | 3.84E-07 | 0.795256341 | 0.72794215 | 0.8687952 | SKN_NEX |
| ZSWIM7 | PD | 482730 | rs4239134 | -0.184401229 | 0.036321454 | 3.84E-07 | 0.831602074 | 0.77445844 | 0.89296207 | LIVER |
| ZSWIM7 | PD | 482730 | rs4792712 | -0.348322034 | 0.068608885 | 3.84E-07 | 0.705871525 | 0.61705603 | 0.80747061 | LUNG |
| ZSWIM7 | PD | 482730 | rs4792712 | -0.229090774 | 0.04512394 | 3.84E-07 | 0.795256341 | 0.72794215 | 0.8687952 | SKN_NEX |

**Supplementary Table 11. Cytobands significantly enriched for the 79 causal genes**

| ID | Name | p-value | q-value Bonferroni | Hit Count in Query List | Hit Count in Genome | Hit in Query List |
|---|---|---|---|---|---|---|
| 17q21.31 | 17q21.31 | 2.66E-24 | 1.17E-22 | 13 | 82 | *LINC02210, MAPT-IT1, MAPK8IP1P1, ARL17B, LRRC37A, KANSL1, KANSL1-AS1, LRRC37A4P, DND1P1, MAPK8IP1P2, ARHGAP27, PLEKHM1, ARL17A* |
| chr17q21 | Ensembl 110 genes in cytogenetic band chr17q21 | 1.03E-18 | 4.53E-17 | 16 | 476 | *LINC02210, MAPT-IT1, MAPK8IP1P1, ARL17B, LRRC37A, KANSL1, KANSL1-AS1, LRRC37A4P, NSF, MAPT, WNT3, DND1P1, MAPK8IP1P2, ARHGAP27, PLEKHM1, ARL17A* |
| 16p11.2 | 16p11.2 | 6.52E-14 | | 10 | 191 | *ZNF668, HSD3B7, VKORC1, STX4, PRSS53, KAT8, BCKDK, PRSS36, STX1B, ZNF646* |

| ID | Name | p-value | q-value Bonferroni | Hit Count in Query List | Hit Count in Genome | Hit in Query List |
|---|---|---|---|---|---|---|
| chr16p11 | Ensembl 110 genes in cytogenetic band chr16p11 | 7.21E-14 | 3.17E-12 | 11 | 278 | *ZNF668, HSD3B7, VKORC1, STX4, PRSS53, KAT8, BCKDK, RNF40, PRSS36, STX1B, ZNF646* |
| 5q12.1 | 5q12.1 | 4.02E-06 | 1.77E-04 | 3 | 24 | *ERCC8, SMIM15, ELOVL7* |
| 4q21.1 | 4q21.1 | 1.52E-05 | 6.70E-04 | 3 | 37 | *STBD1, FAM47E, CCDC158* |
| 4p15 | 4p15 | 3.40E-05 | 1.50E-03 | 2 | 7 | *BST1, CD38* |
| 17p12 | 17p12 | 3.57E-05 | 1.57E-03 | 3 | 49 | *ADORA2B, ZSWIM7, TTC19* |
| chr17p12 | Ensembl 110 genes in cytogenetic band chr17p12 | 8.35E-05 | 3.67E-03 | 3 | 65 | *ADORA2B, ZSWIM7, TTC19* |
| chr5q12 | Ensembl 110 genes in cytogenetic band chr5q12 | 8.74E-05 | 3.84E-03 | 3 | 66 | *ERCC8, SMIM15, ELOVL7* |
| 4p16.3 | 4p16.3 | 1.85E-04 | 8.16E-03 | 3 | 85 | *TMEM175, DGKQ, IDUA* |
| chr4p16 | Ensembl 110 genes in cytogenetic band chr4p16 | 1.98E-04 | 8.69E-03 | 4 | 222 | *GAK, TMEM175, DGKQ, IDUA* |
| chr7p15 | Ensembl 110 genes in cytogenetic band chr7p15 | 3.08E-04 | 1.36E-02 | 3 | 101 | *GPNMB, MALSU1, AK3P3* |
| chr4q21 | Ensembl 110 genes in cytogenetic band chr4q21 | 6.89E-04 | 3.03E-02 | 3 | 133 | *STBD1, FAM47E, CCDC158* |
| chr4p15 | Ensembl 110 genes in cytogenetic band chr4p15 | 7.19E-04 | 3.16E-02 | 3 | 135 | *BST1, CD38, FAM200B* |

**Supplementary Table 12. PD putative causal genes with consistent and varying direction of effects (odds ratios) across multiple issues**

| exposure | AD_COR | AR_AO | AR_CO | SKN_EX | SKN_NEX | BLOOD | LIVER | LUNG | OR across tissues | Direction across tissues |
|---|---|---|---|---|---|---|---|---|---|---|
| *AC005082.12* | NA | 0.7807 | NA | NA | 0.8145 | NA | 0.7640 | 0.7850 | <1 | consistent |
| *ADORA2B* | 1.6021 | 1.1255 | 1.1433 | 1.4570 | 1.4631 | 1.4328 | 1.1359 | 1.4457 | >1 | consistent |
| *ARHGAP27* | 0.5427 | NA | NA | 0.6397 | 0.6133 | NA | NA | NA | <1 | consistent |
| *BCKDK* | NA | NA | NA | 3.2071 | NA | 2.0833 | NA | NA | >1 | consistent |
| *DGKQ* | NA | 0.8066 | 0.7814 | 0.6567 | 0.6460 | NA | NA | NA | <1 | consistent |
| *DND1P1* | NA | 0.8282 | 0.8341 | 0.8326 | 0.8283 | NA | 0.8239 | NA | <1 | consistent |
| *FAM200B* | 1.2464 | 1.5188 | NA | NA | NA | NA | NA | NA | >1 | consistent |
| *FAM47E* | NA | 0.8450 | 0.8473 | 0.8559 | 0.8463 | NA | 0.8717 | NA | <1 | consistent |
| *GAK* | NA | NA | NA | 2.6105 | 1.7611 | NA | NA | 1.6636 | >1 | consistent |
| *GPNMB* | 1.1666 | 1.5344 | NA | 1.4683 | 1.5355 | 1.6881 | 1.4204 | NA | >1 | consistent |
| *IDUA* | NA | 0.5699 | 0.7381 | NA | NA | NA | NA | 0.7144 | <1 | consistent |
| *IGSF9B* | NA | 0.7283 | 0.6816 | NA | NA | 0.6725 | NA | 0.7573 | <1 | consistent |
| *KANSL1* | 1.1789 | NA | NA | NA | NA | NA | 1.2311 | 1.2147 | >1 | consistent |
| *KANSL1-AS1* | NA | 0.8186 | 0.8214 | 0.8277 | 0.8342 | 0.8269 | 0.8238 | 0.8364 | <1 | consistent |
| *KAT8* | 0.8209 | 0.8175 | 0.7750 | 0.8138 | 0.7952 | NA | 0.7456 | 0.7468 | <1 | consistent |
| *KLHL7-AS1* | 0.9206 | 0.9175 | 0.9115 | 0.9187 | 0.9225 | NA | 0.8980 | 0.9088 | <1 | consistent |
| *LRRC37A4P* | NA | 1.2073 | 1.2115 | 1.2347 | 1.2373 | 1.2397 | 1.2505 | NA | >1 | consistent |
| *MAPK8IP1P1* | NA | NA | NA | 0.8139 | 0.8180 | 0.8067 | 0.7946 | 0.8137 | <1 | consistent |
| *MAPK8IP1P2* | NA | 0.8041 | 0.8131 | 0.8123 | 0.8127 | 0.8122 | 0.8408 | 0.8053 | <1 | consistent |
| *MAPT* | NA | NA | NA | 0.6230 | 0.5924 | NA | NA | 0.6439 | <1 | consistent |
| *MMRN1* | 1.4330 | NA | NA | 2.9940 | NA | NA | 1.9615 | NA | >1 | consistent |
| *NUPL2* | 1.1556 | 1.2814 | 1.2197 | 1.9460 | NA | 1.7276 | NA | 1.2957 | >1 | consistent |
| *RAB29* | 1.5179 | 1.2172 | 1.2244 | 1.2632 | 1.2685 | 1.3674 | 1.3241 | 1.2384 | >1 | consistent |
| *RNF40* | 0.6490 | NA | NA | NA | NA | 0.5902 | 0.5589 | 0.5310 | <1 | consistent |
| *RP11-115D19.1* | NA | NA | NA | 0.5149 | 0.5447 | NA | NA | NA | <1 | consistent |
| *RP11-115L11.1* | NA | 0.6950 | NA | 0.6222 | NA | NA | NA | NA | <1 | consistent |

| exposure | AD_COR | AR_AO | AR_CO | SKN_EX | SKN_NEX | BLOOD | LIVER | LUNG | OR across tissues | Direction across tissues |
|---|---|---|---|---|---|---|---|---|---|---|
| *RP11-196G11.2* | 0.7597 | 0.8082 | 0.7847 | 0.8260 | 0.8586 | 0.7642 | 0.7267 | 0.8335 | <1 | consistent |
| *RP11-196G11.3* | NA | NA | NA | NA | 0.7286 | NA | 0.8896 | NA | <1 | consistent |
| *RP11-259G18.1* | NA | 0.7225 | 0.6011 | 0.7427 | 0.7365 | NA | NA | 0.5131 | <1 | consistent |
| *RP11-259G18.3* | NA | 0.8226 | 0.8191 | 0.8194 | 0.8344 | 0.8267 | 0.8278 | 0.8260 | <1 | consistent |
| *RP11-707O23.1* | NA | 0.8098 | NA | 0.7931 | 0.8004 | NA | 0.8161 | NA | <1 | consistent |
| *TMEM163* | NA | 1.2900 | 1.3203 | NA | NA | NA | NA | 2.0558 | >1 | consistent |
| *TMEM175* | 0.7535 | 0.5853 | 0.6769 | 0.5389 | 0.5337 | NA | NA | 0.5881 | <1 | consistent |
| *TTC19* | 0.6124 | 0.6925 | NA | 0.7916 | 0.7830 | 0.7286 | 0.8034 | 0.7911 | <1 | consistent |
| *VKORC1* | NA | NA | NA | 0.6436 | 0.6177 | 2.1679 | 0.9147 | NA | <1 | consistent |
| *WNT3* | NA | 0.7318 | 0.6818 | NA | NA | 0.5706 | NA | 0.6166 | <1 | consistent |
| *ZNF646* | NA | NA | NA | 0.6852 | 0.6999 | NA | 0.7538 | NA | <1 | consistent |
| *ZNF668* | NA | NA | NA | 1.7014 | NA | 1.3303 | NA | 1.6400 | >1 | consistent |
| *ELOVL7* | NA | NA | NA | NA | 0.3783 | 2.1974 | NA | NA | <1 & >1 | varying |
| *HSD3B7* | NA | NA | NA | 0.7935 | 0.7731 | 1.6344 | 0.7022 | NA | <1 & >1 | varying |
| *LINC02210* | 0.6660 | 0.8325 | 0.8224 | 0.8180 | 0.8089 | NA | 1.8258 | 0.8108 | <1 & >1 | varying |
| *PLEKHM1* | NA | NA | NA | NA | NA | 30.2364 | NA | 0.4549 | <1 & >1 | varying |
| *PRSS53* | NA | 1.8090 | NA | 0.6883 | NA | NA | 0.8816 | NA | <1 & >1 | varying |
| *RP11-1072A3.3* | NA | NA | NA | 0.6402 | NA | 1.6362 | NA | NA | <1 & >1 | varying |
| *SNCA* | NA | NA | NA | NA | 2.3922 | 0.1771 | NA | NA | <1 & >1 | varying |
| *STX1B* | NA | 0.8308 | 0.7449 | 0.5966 | 0.5557 | 1.6180 | NA | NA | <1 & >1 | varying |
| *STX4* | 1.5436 | NA | NA | 0.3478 | NA | NA | NA | NA | <1 & >1 | varying |
| *ZSWIM7* | 1.1254 | 1.2963 | NA | 0.8179 | 0.7953 | NA | 0.8316 | 0.7059 | <1 & >1 | varying |

**Supplementary Table 13. Gene ontology (Biological processes) enriched for the PD causal genes**

| p_value | precision | recall | term_id | term_name | intersection |
|---|---|---|---|---|---|
| 0.0034 | 0.0714 | 0.3000 | GO:0001921 | positive regulation of receptor recycling | *RAB29,NSF,SNCA* |
| 0.0115 | 0.0714 | 0.1667 | GO:0001919 | regulation of receptor recycling | *RAB29,NSF,SNCA* |
| 0.0124 | 0.1190 | 0.0385 | GO:0006109 | regulation of carbohydrate metabolic process | *SLC45A3,DGKQ,BCKDK,NCOR1,SNCA* |
| 0.0165 | 0.1190 | 0.0282 | GO:0042180 | cellular ketone metabolic process | *SLC45A3,DGKQ,VKORC1,BCKDK,NCOR1* |
| 0.0165 | 0.0476 | 0.2857 | GO:1903748 | negative regulation of establishment of protein localization to mitochondrion | *LRRK2,MAPT* |
| 0.0165 | 0.0952 | 0.0396 | GO:0090174 | organelle membrane fusion | *STX4,STX1B,SNCA,PLEKHM1* |
| 0.0165 | 0.0476 | 0.2857 | GO:0070493 | thrombin-activated receptor signaling pathway | *DGKQ,SNCA* |
| 0.0165 | 0.1190 | 0.0321 | GO:0061025 | membrane fusion | *STX4,NSF,STX1B,SNCA,PLEKHM1* |
| 0.0165 | 0.0476 | 0.4000 | GO:0048312 | intracellular distribution of mitochondria | *LRRK2,MAPT* |
| 0.0165 | 0.3095 | 0.0087 | GO:0044281 | small molecule metabolic process | *CD38,IDUA,SLC45A3,DGKQ,LRRK2,VKORC1,HSD3B7,BCKDK, ELOVL7,NCOR1,SNCA,MCCC1,FDFT1* |
| 0.0165 | 0.1429 | 0.0211 | GO:1990778 | protein localization to cell periphery | *STX4,RAB29,NSF,STX1B,GAK,SNCA* |
| 0.0165 | 0.0952 | 0.0392 | GO:2000377 | regulation of reactive oxygen species metabolic process | *LRRK2,MAPT,SNCA,BST1* |
| 0.0165 | 0.0714 | 0.0833 | GO:0001881 | receptor recycling | *RAB29,NSF,SNCA* |
| 0.0165 | 0.0952 | 0.0404 | GO:0006906 | vesicle fusion | *STX4,STX1B,SNCA,PLEKHM1* |
| 0.0165 | 0.1667 | 0.0191 | GO:0005975 | carbohydrate metabolic process | *IDUA,SLC45A3,DGKQ,BCKDK,NCOR1,SNCA,STBD1* |
| 0.0168 | 0.1429 | 0.0190 | GO:0016050 | vesicle organization | *STX4,RAB29,STX1B,SNCA,BACE2,PLEKHM1* |
| 0.0185 | 0.2143 | 0.0111 | GO:0001775 | cell activation | *CD38,GPNMB,STX4,RAB29,MMRN1,DGKQ,MAPT,SNCA, BST1* |
| 0.0185 | 0.8095 | 0.0039 | GO:0044237 | cellular metabolic process | *RNF40,CD38,ZSWIM7,GPNMB,STX4,KANSL1,KAT8,IDUA, SLC45A3,DGKQ,WNT3,FAM47E,STX1B,LRRK2,IRF2BP1, ZNF646,GAK,VKORC1,MAPT,ZNF668,HSD3B7,BCKDK, ELOVL7,NCOR1,SNCA,BACE2,MCCC1,PLEKHM1,BST1, MALSU1,ERCC8,NUCKS1,FDFT1,STBD1* |
| 0.0185 | 0.0952 | 0.0364 | GO:0010565 | regulation of cellular ketone metabolic process | *SLC45A3,DGKQ,BCKDK,NCOR1* |
| 0.0210 | 0.0952 | 0.0342 | GO:0099504 | synaptic vesicle cycle | *STX1B,LRRK2,GAK,SNCA* |
| 0.0227 | 0.0952 | 0.0331 | GO:0099003 | vesicle-mediated transport in synapse | *STX1B,LRRK2,GAK,SNCA* |

| p_value | precision | recall | term_id | term_name | intersection |
| --- | --- | --- | --- | --- | --- |
| 0.0265 | 0.8571 | 0.0037 | GO:0008152 | metabolic process | *RNF40,CD38,ZSWIM7,GPNMB,STX4,KANSL1,KAT8,RAB29, NSF,IDUA,SLC45A3,DGKQ,WNT3,FAM47E,STX1B,LRRK2, IRF2BP1,ZNF646,GAK,VKORC1,MAPT,ZNF668,HSD3B7, BCKDK,ELOVL7,NCOR1,SNCA,BACE2,MCCC1,PLEKHM1, BST1,MALSU1,ERCC8,NUCKS1,FDFT1,STBD1* |
| 0.0279 | 0.0476 | 0.1667 | GO:0048311 | mitochondrion distribution | *LRRK2,MAPT* |
| 0.0279 | 0.0714 | 0.0526 | GO:0043112 | receptor metabolic process | *RAB29,NSF,SNCA* |
| 0.0279 | 0.0952 | 0.0299 | GO:0048284 | organelle fusion | *STX4,STX1B,SNCA,PLEKHM1* |
| 0.0279 | 0.6190 | 0.0045 | GO:0019222 | regulation of metabolic process | *RNF40,CD38,GPNMB,STX4,KANSL1,KAT8,RAB29,NSF, SLC45A3,DGKQ,WNT3,FAM47E,STX1B,LRRK2,IRF2BP1, ZNF646,MAPT,ZNF668,BCKDK,NCOR1,SNCA,BACE2,BST1, MALSU1,ERCC8,NUCKS1* |
| 0.0279 | 0.8333 | 0.0038 | GO:0071704 | organic substance metabolic process | *RNF40,CD38,ZSWIM7,GPNMB,STX4,KANSL1,KAT8,RAB29, NSF,IDUA,SLC45A3,DGKQ,WNT3,FAM47E,STX1B,LRRK2, IRF2BP1,ZNF646,GAK,VKORC1,MAPT,ZNF668,HSD3B7, BCKDK,ELOVL7,NCOR1,SNCA,BACE2,MCCC1,BST1,MALSU1, ERCC8,NUCKS1,FDFT1,STBD1* |
| 0.0279 | 0.1190 | 0.0205 | GO:0062012 | regulation of small molecule metabolic process | *SLC45A3,DGKQ,BCKDK,NCOR1,SNCA* |
| 0.0279 | 0.0714 | 0.0517 | GO:0016079 | synaptic vesicle exocytosis | *STX1B,LRRK2,SNCA* |
| 0.0300 | 0.0476 | 0.1538 | GO:0016082 | synaptic vesicle priming | *STX1B,SNCA* |
| 0.0309 | 0.0714 | 0.0469 | GO:0051588 | regulation of neurotransmitter transport | *STX1B,LRRK2,SNCA* |
| 0.0309 | 0.1190 | 0.0191 | GO:0006887 | exocytosis | *STX4,NSF,STX1B,LRRK2,SNCA* |
| 0.0309 | 0.0952 | 0.0270 | GO:0017157 | regulation of exocytosis | *STX4,NSF,LRRK2,SNCA* |
| 0.0309 | 0.0714 | 0.0469 | GO:0010906 | regulation of glucose metabolic process | *SLC45A3,DGKQ,BCKDK* |
| 0.0309 | 0.1190 | 0.0190 | GO:0006979 | response to oxidative stress | *STX4,LRRK2,MAPT,SNCA,ERCC8* |
| 0.0381 | 0.0952 | 0.0250 | GO:0045055 | regulated exocytosis | *STX4,STX1B,LRRK2,SNCA* |
| 0.0408 | 0.5714 | 0.0045 | GO:0060255 | regulation of macromolecule metabolic process | *RNF40,CD38,GPNMB,STX4,KANSL1,KAT8,RAB29,NSF,DGKQ, WNT3,FAM47E,STX1B,LRRK2,IRF2BP1,ZNF646,MAPT, ZNF668,NCOR1,SNCA,BACE2,BST1,MALSU1,ERCC8,NUCKS1* |
| 0.0408 | 0.0952 | 0.0235 | GO:0072593 | reactive oxygen species metabolic process | *LRRK2,MAPT,SNCA,BST1* |
| 0.0408 | 0.1190 | 0.0169 | GO:0045936 | negative regulation of phosphate metabolic process | *DGKQ,LRRK2,MAPT,NCOR1,SNCA* |
| 0.0408 | 0.0476 | 0.1111 | GO:0009083 | branched-chain amino acid catabolic process | *BCKDK,MCCC1* |
| 0.0408 | 0.1190 | 0.0168 | GO:0010563 | negative regulation of phosphorus metabolic process | *DGKQ,LRRK2,MAPT,NCOR1,SNCA* |
| 0.0408 | 0.1190 | 0.0170 | GO:0010975 | regulation of neuron projection development | *RAB29,STX1B,LRRK2,GAK,MAPT* |

| p_value | precision | recall | term_id | term_name | intersection |
|---|---|---|---|---|---|
| 0.0408 | 0.0714 | 0.0405 | GO:0050848 | regulation of calcium-mediated signaling | *LRRK2,MAPT,BST1* |
| 0.0408 | 0.5714 | 0.0045 | GO:0031323 | regulation of cellular metabolic process | *RNF40,CD38,GPNMB,STX4,KANSL1,KAT8,SLC45A3,DGKQ, WNT3,FAM47E,STX1B,LRRK2,IRF2BP1,ZNF646,MAPT, ZNF668,BCKDK,NCOR1,SNCA,BACE2,BST1,MALSU1,ERCC8, NUCKS1* |
| 0.0435 | 0.0952 | 0.0227 | GO:0034599 | cellular response to oxidative stress | *STX4,LRRK2,MAPT,SNCA* |
| 0.0447 | 0.4048 | 0.0055 | GO:0009893 | positive regulation of metabolic process | *RNF40,CD38,GPNMB,STX4,KANSL1,KAT8,RAB29,NSF, SLC45A3,DGKQ,WNT3,FAM47E,LRRK2,MAPT,SNCA,ERCC8, NUCKS1* |
| 0.0447 | 0.1429 | 0.0130 | GO:0120035 | regulation of plasma membrane bounded cell projection organization | *RAB29,STX1B,LRRK2,GAK,MAPT,PLEKHM1* |
| 0.0451 | 0.0476 | 0.1000 | GO:0090322 | regulation of superoxide metabolic process | *MAPT,BST1* |
| 0.0469 | 0.3810 | 0.0056 | GO:0010604 | positive regulation of macromolecule metabolic process | *RNF40,CD38,GPNMB,STX4,KANSL1,KAT8,RAB29,NSF,DGKQ, WNT3,FAM47E,LRRK2,MAPT,SNCA,ERCC8,NUCKS1* |
| 0.0474 | 0.1429 | 0.0126 | GO:0031344 | regulation of cell projection organization | *RAB29,STX1B,LRRK2,GAK,MAPT,PLEKHM1* |
| 0.0474 | 0.0476 | 0.0952 | GO:1902774 | late endosome to lysosome transport | *LRRK2,PLEKHM1* |
| 0.0481 | 0.1429 | 0.0125 | GO:0044283 | small molecule biosynthetic process | *SLC45A3,DGKQ,HSD3B7,ELOVL7,SNCA,FDFT1* |
| 0.0481 | 0.0952 | 0.0211 | GO:1901617 | organic hydroxy compound biosynthetic process | *DGKQ,HSD3B7,SNCA,FDFT1* |
| 0.0481 | 0.0714 | 0.0349 | GO:0010977 | negative regulation of neuron projection development | *RAB29,STX1B,GAK* |
| 0.0495 | 0.1429 | 0.0122 | GO:0061919 | process utilizing autophagic mechanism | *KAT8,LRRK2,MAPT,SNCA,PLEKHM1,STBD1* |
| 0.0495 | 0.1429 | 0.0122 | GO:0006914 | autophagy | *KAT8,LRRK2,MAPT,SNCA,PLEKHM1,STBD1* |

**Supplementary Table 14. Changes in *PLEKHM1* transcripts expression associated with risk or protection for PD**

| Gene | Isoform | Ensembl transcript biotype | Tissue Comparison | Fold change in blood |
|---|---|---|---|---|
| *PLEKHM1* | ENST00000580404.5 | Protein coding CDS not defined | lung | 0.65 |
| | ENST00000430334.7 | Protein-coding | lung | 0.49 |
| | ENST00000581448.5 | Nonsense mediated decay | lung | 0.42 |
| | ENST00000582035.1 | - | lung | 0.89 |
| | ENST00000591580.1 | Protein-coding | lung | 0.59 |
| | ENST00000585506.1 | Retained intron | lung | 0.63 |
| | ENST00000579197.5 | Nonsense mediated decay | lung | 2.27 |
| | ENST00000586084.1 | Nonsense mediated decay | lung | 0.13 |
| | ENST00000590991.1 | Nonsense mediated decay | lung | 0.26 |
| | ENST00000579131.5 | Protein coding CDS not defined | lung | 0.48 |
| | ENST00000586562.5 | Nonsense mediated decay | lung | 0.76 |
| | ENST00000581932.2 | - | lung | 0.69 |
| | ENST00000584420.1 | Protein-coding | lung | Total loss |
| | ENST00000580205.1 | Protein coding CDS not defined | lung | 0.38 |
